# Programmed hierarchical patterning of bacterial populations

**DOI:** 10.1038/s41467-018-03069-3

**Published:** 2018-02-22

**Authors:** Christian R. Boehm, Paul K. Grant, Jim Haseloff

**Affiliations:** 1 Department of Plant Sciences, University of Cambridge, Downing Street, Cambridge, CB2 3EA UK; 2 Microsoft Research, 21 Station Road, Cambridge, CB1 2FB UK; 3 Present Address: Max Planck Institute of Molecular Plant Physiology, Am Mühlenberg 1, 14476 Potsdam, Germany

## Abstract

Modern genetic tools allow the dissection and emulation of fundamental mechanisms shaping morphogenesis in multicellular organisms. Several synthetic genetic circuits for control of multicellular patterning have been reported to date. However, hierarchical induction of gene expression domains has received little attention from synthetic biologists, despite its importance in biological self-organization. Here we report a synthetic genetic system implementing population-based AND-logic for programmed autonomous induction of bacterial gene expression domains. We develop a ratiometric assay for bacteriophage T7 RNA polymerase activity and use it to systematically characterize different intact and split enzyme variants. We then utilize the best-performing variant to build a three-color patterning system responsive to two different homoserine lactones. We validate the AND gate-like behavior of this system both in cell suspension and in surface culture. Finally, we use the synthetic circuit in a membrane-based spatial assay to demonstrate programmed hierarchical patterning of gene expression across bacterial populations.

## Abstract (web summary)

Biological self-organization uses hierarchical induction of gene expression but this has not been exploited by synthetic biology. Here the authors use an AND gate based on a split T7 RNA polymerase to organize programmed hierarchical patterning of gene expression across a bacterial population.

## Introduction

The hierarchical organization of multicellular organisms builds on mechanical and chemical interactions. Cells sense cues in their environment and modulate both their metabolism and communication with neighbors. At the population level, the interplay of multiple processes across time and space can lead to the emergence of self-organization through mechanisms such as symmetry breaking, domain induction, and boundary formation^1^. Despite the advances made in the biological sciences over past decades, many of the mechanisms underlying complex patterning and morphogenesis remain elusive.

In efforts to explain multicellular patterning, two types of models have been pre-eminent to date: the reaction-diffusion (RD) model proposed by Alan Turing^2^, and the positional information (PI) model (also known as the “French flag model”) originating from Lewis Wolpert^3^. RD-type systems are characterized by self-organized spatial patterns emerging from interacting positive- and negative-feedback loops in response to two diffusible morphogens. In contrast, PI-type models employ a single predefined morphogen gradient, which is interpreted by receiving cells according to the local concentration of the morphogen. Though the two models are conceptually distinct, they are not necessarily mutually exclusive^4^.

Empowered by modern genetic techniques, molecular biologists are now striving to not only dissect developmental processes, but to exploit their modularity to customize the design of living systems^5^. Biological self-organization is a powerful tool for bioprocessing and remediation in tailored microbial consortia, for sustainable bioproduction in novel plant compartments, or for applications in regenerative medicine enabled by engineered vertebrate tissues. A fundamental requirement for harnessing this potential is control over the differentiation of cell types to create domains of gene expression in spatially organized patterns. To date, several synthetic biological circuits capable of multicellular patterning have been reported, predominantly implemented either by RD-type^6,7^ or by PI-type^8–11^ mechanisms.

However, mechanisms for hierarchical patterning have received little attention from synthetic biologists to date. In various examples of morphogenesis, such as vulval induction in nematodes^12^, the ABC model of flower development^13^, or mesoderm induction in vertebrates^14^, we can observe nested domains of gene expression. To emulate biological self-organization on this level of complexity, we require control over hierarchical induction of new domains within existing patterns.

Approaching this challenge, we report the implementation of a synthetic genetic circuit that controls emergence of a new domain of gene expression at the interface of existing bacterial populations. To the best of our knowledge, this is the first description of a synthetic genetic circuit implementing AND-logic for autonomous hierarchical patterning at the population scale. A previously reported population-based edge detector has implemented AND (NOT (NOT)) logic in response to light projected through a mask^15^. In contrast, the circuit introduced here is designed to establish two layers of patterning in the absence of an externally defined spatial input.

As intercellular signals mediating domain induction, we utilize the diffusible small molecule signals *N*-(3-oxohexanoyl)-l-homoserine lactone (3OC6HSL) and *N*-(3-oxododecanoyl)-l-homoserine lactone (3OC12HSL). These compounds are derived from the bacterial quorum-sensing systems from *Vibrio fischeri*^16^ and *Pseudomonas aeruginosa*^17^, respectively. Both systems employ a single biosynthetic enzyme to produce a diffusible signal (LuxI/LasI), and a single receiver protein (LuxR/LasR) to activate transcription of a target gene controlled by their cognate promoters (P_Lux_/P_Las_) upon signal binding^18^. The two signaling systems have been previously combined as part of synthetic circuits, but by default exhibit significant levels of crosstalk^19–24^. Overcoming this complication, we have recently reported an intercellular signaling system minimizing crosstalk between 3OC6HSL and 3OC12HSL in the same cell^24^.

**Fig. 1:**
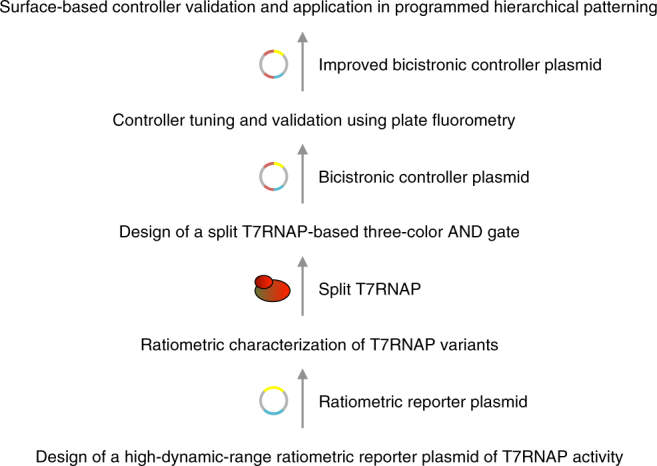
Design workflow of a genetic circuit for synthetic hierarchical patterning. The resulting synthetic circuit autonomously mediates the programmed emergence of a gene expression domain at the interface of two bacterial populations

In this work, we combined this improved intercellular signaling system with the transcriptional output from an orthogonal split bacteriophage T7 RNA polymerase (T7RNAP)^25–29^ to establish hierarchical induction of gene expression domains at the population scale. A first layer of patterning was established via promoters that respond to the two different homoserine lactones (HSLs) and induce production of cyan (CFP) and yellow fluorescent protein (YFP), respectively. In addition, each of the two HSLs induced expression of one half of the split T7RNAP protein. This was used to generate a second layer of patterning, where RNA polymerase activity was limited to where the two diffusible signals coincide, and was marked by expression of red fluorescent protein (RFP) from a T7 promoter. In order to construct this system, we characterized systematically the activity of different *T7RNAP* genes using a ratiometric strategy to identify the split variant of highest dynamic range. We then implemented a synthetic three-color AND gate based on split T7RNAP responsive to 3OC6HSL and 3OC12HSL in both cell suspensions and surface cultures of *Escherichia coli*. Finally, we demonstrated that the synthetic circuit autonomously mediated programmed emergence of a new gene expression domain at the interface of two bacterial populations (Fig. 1).

## Results

### Design of a ratiometric reporter for T7RNAP activity

Rational design of a synthetic circuit generating desired multicellular behavior requires a quantitative understanding of its core genetic components. To increase robustness in measurements of activity from the T7 promoter, we extended a previously reported ratiometric strategy^30,31^ to T7RNAP-driven gene expression: in our ratiometric reporter plasmids, the YFP variant *mVenus*^32^ served as a primary reporter of T7RNAP activity. In addition, the ratiometric reporter plasmids encoded a CFP variant *mTurquoise2*^33^ that was constitutively expressed under control of reference promoter P_J23101_ and reference RBS_B0034_ (MIT Registry of Standard Biological Parts). Relative promoter activity could be expressed as the average YFP over CFP fluorescence intensity per cell during exponential growth phase^30,31^. This indicator was chosen in order to correct for variation in cellular gene expression capacity due to environmental conditions.

We designed a small library of plasmid reporters for T7RNAP activity to identify a combination of promoter, 5′-UTR, and copy number capable of supporting high dynamic range in reporter induction. This library embraced all combinations of (i) the wild-type T7 promoter P_T7_ (refs. ^34–36^) or a mutant promoter P_T7_(-3G) (~20% activity of the consensus sequence)^37^, (ii) the 5′-UTR from bacteriophage T7 gene 10 RBS_T7g10_ (ref. ^38^) or a synthetic 5′-UTR embracing the reference RBS_B0034_ (MIT Registry of Standard Biological Parts), and (iii) the high copy number backbone pSB1A3 (pMB1 ori, MIT Registry of Standard Biological Parts) or the low copy number backbone pSB4A5 (pSC101 ori, MIT Registry of Standard Biological Parts). Notably, these plasmids included a LacI generator cassette to reduce leaky expression of *T7RNAP* from the genome of *T7 Express E*. *coli*.

**Fig. 2:**
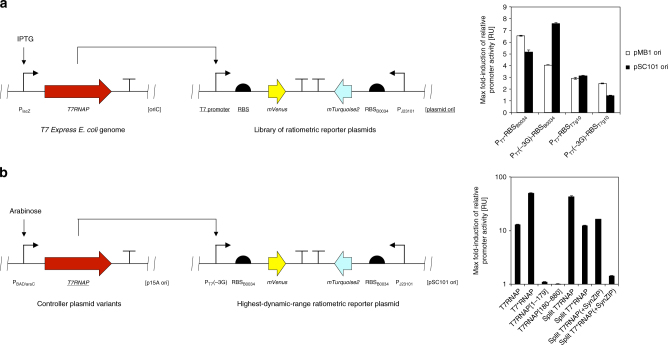
Ratiometric characterization of T7RNAP activity. **a** Comparison of different ratiometric reporter plasmids of T7RNAP activity. Ratiometric reporter plasmids combining different variants of the T7 promoter, 5′-UTRs, and origins of replication (underlined) were introduced into *T7 Express E*. *coli*. The maximum fold-induction of relative activity from the T7 promoter in response to a range of IPTG concentrations (see Supplementary Figure 1 for induction curves) was quantified using a plate fluorometer-based assay (see Methods for details), and is reported relative to the absence of inducer. Error bars represent the standard deviation (s.d.) of average values yielded between three biological replicate experiments performed on different days. **b** Ratiometric characterization of intact and split variants of T7RNAP. *TOP10 E*. *coli* were co-transformed by the highest dynamic range ratiometric reporter p4g3VT tested under **a** and different controller plasmids encoding intact or fragmented variants of the *T7RNAP* gene (underlined) under control of the arabinose-inducible P_BAD/araC_ promoter. The maximum fold-induction of relative activity from the T7 promoter in response to a range of arabinose concentrations was quantified as described above (see Supplementary Figure 2 for induction curves), and is reported relative to the absence of inducer. Error bars represent the s.d. of average values yielded between three biological replicate experiments performed on different days

To quantify relative activity from the T7 promoter across the small library of ratiometric reporters outlined above, we introduced the plasmids individually into *T7 Express E*. *coli* encoding a single genomic copy of *T7RNAP* under control of the *lac* operon. After induction of *T7RNAP* expression in transformed *T7 Express E*. *coli* using a range of isopropyl-β-d-thiogalactopyranoside (IPTG) concentrations (Supplementary Figure 1), we performed ratiometric assays in a plate fluorometer to measure CFP and YFP fluorescence intensities and optical density over time for measurement of relative promoter activity (Fig. 2a; see Methods for details). Taking this experimental approach, we found that combining the mutant P_T7_(-3G) promoter with reference RBS_B0034_ on a low copy number backbone produced the highest induction of relative activity from the T7 promoter after addition of inducer (7.58 ± 0.08-fold).

### Ratiometric characterization of T7RNAP variants

Next, we used the reporter plasmid displaying the highest dynamic range in *T7 Express E*. *coli* for measurement of the activity of different intact and split T7RNAP variants. We co-transformed *TOP10 E*. *coli* with the ratiometric reporter plasmid p4g3VT and controller plasmids encoding intact and fragmented *T7RNAP* genes under control of the arabinose-inducible P_BAD/araC_ system^26^. The LacI generator was removed from the ratiometric reporter plasmid p4g3VT_LacI_ for this purpose. Performing ratiometric assays with a range of arabinose concentrations in a plate fluorometer-based format, we confirmed that induction of relative activity from the T7 promoter was negligible for both of the individual N-terminal (T7RNAP[1–179]: 1.10 ± 0.01-fold) and C-terminal (T7RNAP[180-880]: 0.94 ± 0.05-fold) fragments of T7RNAP compared to the intact enzyme (T7RNAP: 12.9 ± 0.3-fold; Fig. 2b). High induction of T7RNAP (i.e. at inducer concentrations exceeding 1 mM arabinose) led to a drop in relative promoter activity (see Supplementary Figure 2). Incorporation of the mutation R632S^39^ into the intact wild-type enzyme increased the maximum induction of relative promoter activity almost by a factor of 4 (T7*RNAP: 50.1 ± 0.9-fold). Furthermore, the inducer concentration required for half maximal activity was increased approximately 30-fold. In contrast to T7RNAP, no inverse correlation between inducer concentration and promoter activity was observed for T7*RNAP up to 50 mM arabinose. Splitting wild-type T7RNAP between residues 179 and 180^25,26^ increased its dynamic range by over a factor of 3 (split T7RNAP: 43 ± 2-fold). In contrast to the intact enzyme, incorporation of the R632S mutation decreased the maximum induction of relative promoter activity in split T7RNAP (split T7*RNAP: 12.3 ± 0.3-fold). Adopting an approach previously taken^27^, we also tested whether the addition of SynZIP protein–protein interaction domains^40,41^ to wild type and mutant (R632S) variants of split T7RNAP increased their dynamic range. Compared to enzyme modifications described above, application of SynZIP domains further increased the inducer concentration required for half maximal activity (i.e. over 100-fold relative to T7RNAP; see Supplementary Figure 2). However, neither modified variant exceeded wild-type split T7RNAP in maximum induction of activity from the T7 promoter (split T7RNAP(+SynZIP): 16.3 ± 0.3-fold; split T7*RNAP(+SynZIP): 1.42 ± 0.04-fold). From the ratiometric assays performed, we concluded that split T7RNAP was the most promising nicked variant to employ in our synthetic patterning circuit.

### Design of an HSL-responsive three-color AND gate

**Fig. 3:**
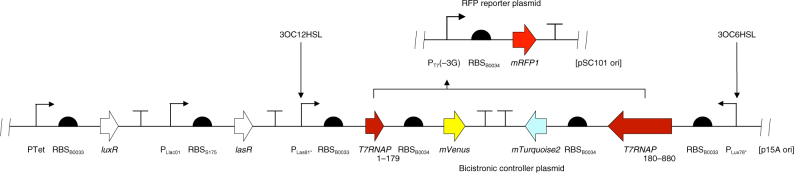
Design of a split T7RNAP-based three-color circuit responsive to HSLs. Schematic representations of the *mRFP1*-expressing reporter of T7RNAP activity p4g3R (see Supplementary Figure 3 for induction curve) and the bicistronic controller plasmid pCRB DRT7VTPLux*500 encoding two bicistronic operons responding to 3OC6HSL and 3OC12HSL by induction of *T7RNAP[180-880]* and *mTurquoise2*, or *T7RNAP*[1–179] and *mVenus*, respectively, are shown

Having validated the activity of split T7RNAP, we utilized this enzyme to design a three-color synthetic transcriptional AND gate. To this end, we assembled a controller plasmid embracing two bicistronic operons composed of *T7RNAP*[1–179] and *mVenus*, and *T7RNAP[180–880]* and *mTurquoise2*, respectively (Fig. 3). The downstream fluorescent proteins were included to serve as visual indicators of expression of the individual T7RNAP fragments in vivo. The two bicistronic operons were controlled by hybrid promoters responsive to either 3OC12HSL (P_Las81*_) or 3OC6HSL (P_Lux76*_), respectively, alongside weak ribosomal binding sites RBS_B0033_ (MIT Registry of Standard Biological Parts). The promoters in question were derived from our previously reported system for orthogonal intercellular signaling^24^, and contained mutations shown to reduce basal activity from the Lux promoter^42^. Cognate receiver proteins LuxR and LasR were constitutively expressed from the vector backbone pR33S175. We also constructed an RFP reporter plasmid of T7RNAP activity p4g3R based on the design principles proven earlier (see Fig. 2a): the *mRFP1* gene was controlled by P_T7_(-3G) and RBS_B0034_ on a low copy number pSC101 backbone. In contrast to ratiometric reporter plasmids, p4g3R lacked the P_J23101_-*mTurquoise2* reference operon. We validated the response of p4g3R to T7RNAP by introduction into *T7 Express E*. *coli* and measuring RFP output in varying concentrations of IPTG (Supplementary Figure 3).

We began construction of the AND gate by testing the behavior of the two individual controller half circuits. For this purpose, we built half-circuit controller plasmids pCRB DRT7VTPLux*500CFP and pCRB DRT7VTPLux*500YFP, lacking either the bicistronic operon P_Las81*_-*T7RNAP*[1–179]-*mVenus* or P_Lux76*_-*T7RNAP[180-880]*-*mTurquoise2*, respectively. We transformed *TOP10 E*. *coli* cells with each half-circuit controller plasmid in addition to the RFP reporter plasmid p4g3R. We measured CFP, YFP, and RFP fluorescence intensities over time as a function of 3OC6HSL and 3OC12HSL concentrations using a plate fluorometer-based assay (see Methods for details). As expected, the half-circuit bicistronic controller plasmid pCRB DRT7VTPLux*500CFP responded to increasing concentrations of 3OC6HSL with increased induction of corrected CFP fluorescence intensity. Similarly, the half-circuit bicistronic controller plasmid pCRB DRT7VTPLux*500YFP responded to increasing concentrations of 3OC12HSL with increased induction of corrected YFP fluorescence intensity (Table 1, Supplementary Figure 4a, b). Induction of corrected RFP intensity from the reporter plasmid was negligible for both half-circuit bicistronic controller plasmids. Under the same assay conditions, the complete bicistronic controller plasmid pCRB DRT7VTPLux*500 produced substantial levels of corrected RFP fluorescence intensity only if exposed to both 3OC6HSL and 3OC12HSL (Supplementary Figure 4c). Notably, we observed a reduction in corrected YFP (up to 29 ± 7%) and CFP (up to 46 ± 9%) fluorescence intensities under conditions of high RFP induction.

**Table 1:** Comparison of a bicistronic controller plasmid and its constitutive half circuits

	Max corrected fluorescence intensity [RFU]		
	CFP	YFP	RFP
pCRB DRT7VTPLux*500CFP	7000 ± 1000	9 ± 9	100 ± 20
pCRB DRT7VTPLux*500YFP	1200 ± 200	5000 ± 1000	150 ± 20
pCRB DRT7VTPLux*500	5000 ± 1000	5000 ± 1000	11,000 ± 3000

*TOP10 E*. *coli* were co-transformed with p4g3R, an *mRFP1*-expressing reporter of T7RNAP activity, and the bicistronic controller plasmid pCRB DRT7VTPLux*500 or one of its constitutive half circuits. In contrast to pCRB DRT7VTPLux*500, pCRB DRT7VTPLux*500CFP and pCRB DRT7VTPLux*500YFP lacked either the bicistronic operon P_Las81*_-*T7RNAP*[1–179]-*mVenus* or P_Lux76*_-*T7RNAP[180-880]*-*mTurquoise2*, respectively. The behavior of bicistronic controller plasmid variants was tested alongside the RFP reporter plasmid under a two-dimensional titration of 3OC6HSL and 3OC12HSL using a plate fluorometer-based assay (see Methods for details). The maximum observed fluorescence intensity, corrected for background signal present in the absence of externally supplied HSLs, is reported. Values shown are the mean of three biological replicate experiments performed on different days, plus or minus their s.d. Activity plots are shown in Supplementary Figure 4. Individual induction curves are shown in Supplementary Figure 5.

**Fig. 4:**
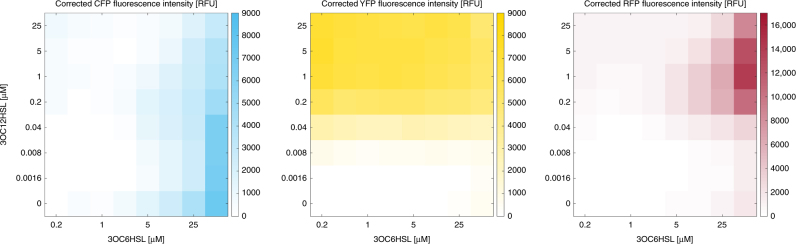
An improved synthetic three-color circuit implementing AND gate-like behavior. The behavior of the high-dynamic-range bicistronic controller plasmid pCRB DRT7VTPLux*250 was tested alongside the RFP reporter plasmid p4g3R under a two-dimensional titration of 3OC6HSL and 3OC12HSL using a plate fluorometer-based assay (see Methods for details). Fluorescence intensity, corrected for background signal present in the absence of externally supplied HSLs, is reported for each condition. Plots show average values from three biological replicate experiments performed on different days. Corresponding s.d. values are shown alongside individual induction curves in Supplementary Figure 8

While measurement of corrected RFP intensity under two-dimensional titration of HSLs showed AND gate-like behavior of our circuit (Supplementary Figure 4c), we sought to further reduce background induction in the absence of 3OC12HSL. Corrected RFP fluorescence intensity in the absence of 3OC6HSL was already low. We replaced RBS_B0033_ (relative strength 500 arbitrary units) regulating expression of *T7RNAP*[1–179] in pCRB DRT7VTPLux*500 by a series of weaker ribosomal binding sites (relative strength 250, 100, and 50 arbitrary units) designed by the Ribosome Binding Site Calculator^43^. We measured the response of the resulting bicistronic controller plasmid variants to increasing concentrations of 3OC12HSL using plate fluorometry. pCRB DRT7VTPLux*250 demonstrated the greatest sensitivity to 3OC12HSL (Supplementary Figures 6 and 7). When we induced the improved three-color circuit composed of bicistronic controller pCRB DRT7VTPLux*250 and reporter plasmid p4g3R with both HSLs, we observed corrected RFP fluorescence intensity of over 8-fold of the maximum background detected in the absence of either input signal (Supplementary Figure 8). The improved synthetic three-color circuit correctly exhibited the behavior of a synthetic transcriptional AND gate responsive to two different HSLs (Fig. 4).

### HSL-mediated patterning of bacterial populations

To test whether our three-color synthetic transcriptional AND gate was responding to HSLs in surface culture as well as in suspension, we employed a previously reported spatial assay based on membranes printed with hydrophobic grids^24^. Bacterial populations were confined to quadrants with well-defined geometry, separated from one another while allowing HSL-mediated communication across the substrate. Following inoculation of membranes with dilute bacterial cultures, changes in CFP, YFP, and RFP fluorescence intensities in response to environmental signals could be monitored across the grid over time using a custom macroscopic imaging system (see Methods for details).

**Fig. 5:**
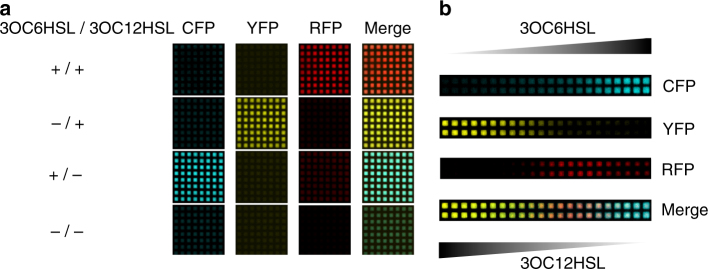
HSL-responsive surface-based patterning of bacterial gene expression. **a** Surface-based circuit behavior in response to HSLs present in the growth medium at uniform concentration. *TOP10 E*. *coli* co-transformed by the RFP reporter plasmid p4g3R and the improved bicistronic controller plasmid pCRB DRT7VTPLux*250 (see Fig. 3) were incubated on membranes printed with hydrophobic ink, placed on minimal agar containing different combinations of 3OC6HSL (25 μM) and 3OC12HSL (1 μM). Images shown were captured at *t* = 1500 min (time relative to start of incubation). Corresponding corrected fluorescence intensities are shown in Supplementary Figure 9. **b** Surface-based AND gate behavior in response to HSL gradients. *TOP10 E*. *coli* cells co-transformed by controller and reporter plasmids as above were incubated on membranes placed on minimal agar lacking supplemented HSLs. Instead, aqueous solutions containing 500 μM 3OC6HSL or 200 μM 3OC12HSL, respectively, were spotted next to the cells on either side and left to diffuse into the bacterial population from opposite directions. Images shown were captured at *t* = 3000 min (time relative to start of incubation). Corrected fluorescence intensities recorded over time are shown in Supplementary Figure 10

First, we uniformly inoculated 64 membrane quadrants with bacteria co-transformed by the RFP reporter plasmid p4g3R and the improved bicistronic controller plasmid pCRB DRT7VTPLux*250. In this experiment, membranes were placed on minimal nutrient agar containing (i) no added HSLs, (ii) 25 μM 3OC6HSL, (iii) 1 μM 3OC12HSL, or (iv) 25 μM 3OC6HSL and 1 μM 3OC12HSL. The concentrations of signaling molecules were chosen to match the condition of highest induction of RFP fluorescence intensity observed in suspension culture (see Fig. 4). Solid culture assays performed at 37 °C as outlined above confirmed that presence of both HSLs was required for substantial induction of corrected RFP fluorescence intensity, suggesting AND gate-like behavior (Fig. 5a). Corrected CFP and YFP fluorescence intensities were reduced to background levels under the condition of co-induction compared to presence of either signaling molecule alone.

Next, we sought to test whether the same synthetic three-color circuit could implement surface-based spatial patterning across a bacterial population in response to gradients of diffusing HSLs. For this purpose, we inoculated rows of quadrants with double-transformed cells on agar lacking supplemented HSLs. At a distance of one quadrant from either end of the inoculated rows, we then spotted solutions of 3OC6HSL (500 μM) or 3OC12HSL (200 μM), and allowed the inducers to diffuse into the bacterial population from opposite directions. Over a course of 50 h, we observed the emergence of a three-color pattern across the bacterial population: with fluorescence of CFP and YFP mirroring the opposite gradients of 3OC6HSL and 3OC12HSL, RFP fluorescence was found to peak near the center of the inoculated rows where the two gradients overlap (Fig. 5b).

### Programmed emergence of a bacterial gene expression domain

Finally, we implemented a greater degree of autonomy in our synthetic three-color patterning system by enabling bacterial cells to produce and secrete their own inducers. The improved bicistronic controller plasmid pCRB DRT7VTPLux*250 and the RFP reporter p4g3R had been characterized both in suspension (see Fig. 4) and in surface culture (see Fig. 5). We introduced a third plasmid into *E*. *coli*: the sender plasmids pSB1C3 I0500 (LuxI/LasI) contained either *luxI* (encoding an enzyme producing 3OC6HSL; ref.^16^) or *lasI* (encoding an enzyme producing 3OC12HSL; ref.^17^) under control of the P_BAD/araC_ promoter system.

**Fig. 6:**
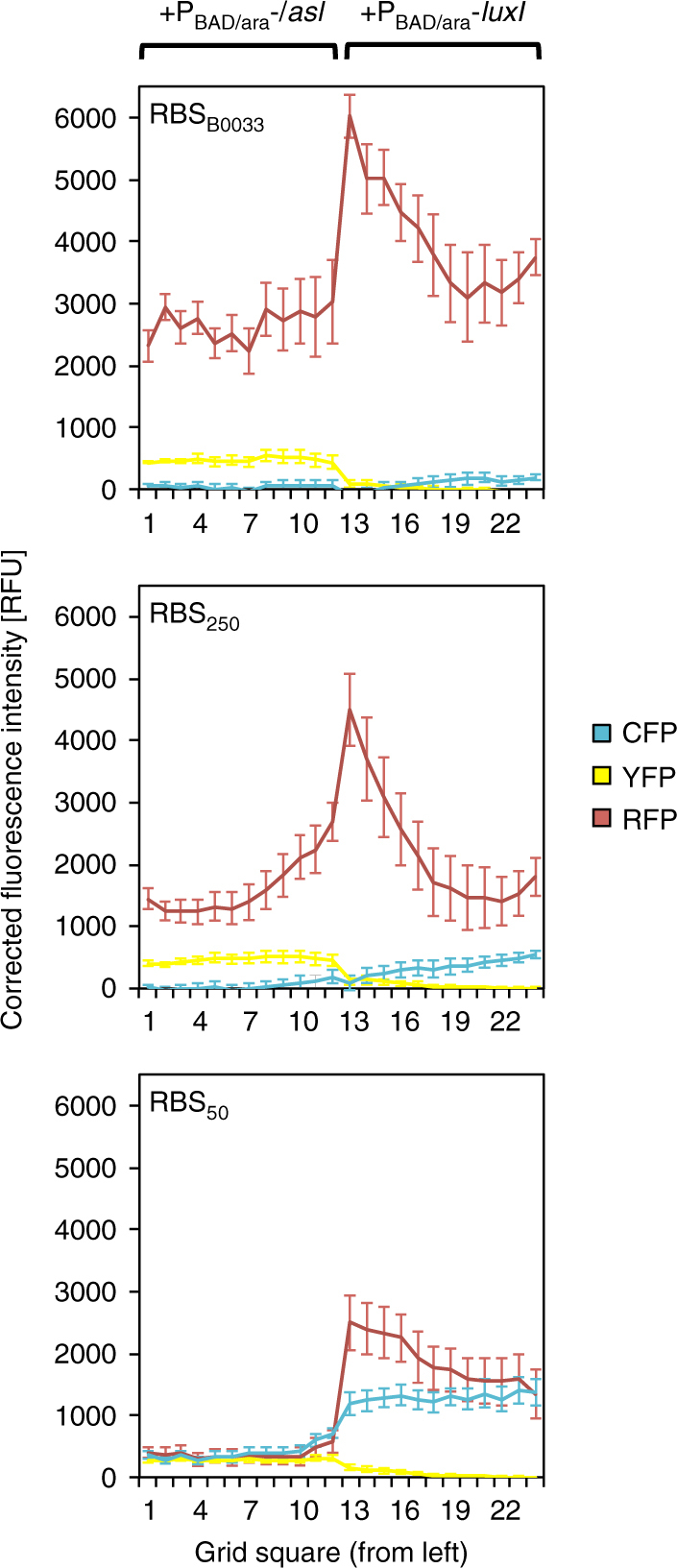
Programmed emergence of an RFP-expressing domain across bacterial populations. *TOP10 E*. *coli* were co-transformed by the RFP reporter plasmid p4g3R, a bicistronic controller plasmid pCRB DRT7VTPLux*(500/250/50), and a sender plasmid pSB1C3 I0500 (LuxI/LasI) encoding either *luxI* or *lasI* under control of the arabinose-inducible P_BAD/araC_ promoter. Adjacent populations of the different triple-transformed cell types were incubated on membranes placed on minimal nutrient agar which has or has not been supplemented with 25 mM arabinose. Fluorescence intensity of each quadrant at *t* = 3000 min (time relative to start of incubation), corrected for background signal present in the absence of arabinose, is plotted against position (genotype boundary between quadrants 12 and 13). Error bars represent the s.d. of average values between the 12 equidistant quadrants on each membrane. Corresponding images captured at *t* = 3000 min are shown in Supplementary Figure 11

Utilizing solid culture assays, we inoculated membrane grids with two adjacent populations of the different triple-transformed cell types: each population contained (i) the RFP reporter, (ii) the improved bicistronic controller, and (iii) either the LuxI or the LasI sender plasmid. In the absence of arabinose, fluorescence intensity was low in all three channels. In the presence of 25 mM arabinose, corrected CFP and YFP fluorescence intensities were substantially induced in cells expressing LuxI and LasI, respectively (Fig. 6). By comparing arabinose-induced populations to HSL standard curves prepared under the same conditions (Supplementary Figure 12), we were able to estimate the effective concentration of HSLs those populations were experiencing. We estimated the effective concentrations of 3OC6HSL and 3OC12HSL in their respective domains of synthesis to be roughly 0.2–1 μM and 1 μM, respectively, at quadrants farthest from the interface. As intended, a symmetrical domain of high RFP activity spontaneously emerged at the interface of 3OC6HSL- and 3OC12HSL-sending cell populations. Our synthetic three-color patterning circuit generated programmed emergence of this new gene expression domain in absence of externally applied signaling gradients, implementing an autonomous mechanism for hierarchical patterning.

To assess the robustness of this behavior, we subjected the suboptimal bicistronic controller plasmids pCRB DRT7VTPLux*500 and pCRB DRT7VTPLux*50 (see Supplementary Figure 6) to the same solid culture experiment. The use of the stronger-than-optimal RBS_B0033_ (pCRB DRT7VTPLux*500) led to slightly increased corrected YFP intensity and reduction of corrected CFP fluorescence intensity by approximately two-thirds compared to the optimal plasmid. Peak corrected RFP fluorescence intensity in this context was one-third higher than that observed using the optimal plasmid but RFP fluorescence in the LuxI-expressing domain (measured in quadrants farthest from the interface) was more than double that of the optimal system (Fig. 6 and Supplementary Figure 11).

Use of the weaker-than-optimal RBS_50_ (pCRB DRT7VTPLux*50) resulted in a one-third reduction in corrected YFP fluorescence intensity and a doubling of corrected CFP fluorescence intensity compared to optimal. Peak corrected RFP fluorescence intensity was reduced almost to half of its original value, with high background activity across the *luxI*-expressing domain (Fig. 6 and Supplementary Figure 11). Comparing the behavior of circuit variants in solid and liquid culture reveals that the variants shown to be suboptimal in plate fluorometer assays (see Supplementary Figure 6) are also suboptimal in membrane-based assays. This highlights the power of modular empirical component optimization in implementing circuits with complex spatial behavior on solid surfaces.

## Discussion

Hierarchical induction of gene expression domains is a key mechanism for patterning in living organisms as they establish their body plan and physical shape^12,14,44^. However, this mechanism has received little attention from synthetic biologists in efforts to emulate developmental processes fundamental to morphogenesis^5–11,24^. In this work, we implemented programmed spatial patterning resembling hierarchical induction in populations of bacterial cells. Our approach was based on a synthetic three-color genetic circuit embracing a split T7RNAP. This enzyme was controlled by two different signaling molecules derived from bacterial quorum sensing (3OC6HSL and 3OC12HSL)^16–18^. The HSLs shaped a first layer of patterning and spatially controlled expression of split T7RNAP, which then established a second layer of patterning by transcriptional activity from its cognate T7 promoter.

In developing our synthetic circuit, we first sought to identify a variant of split T7RNAP exhibiting the highest dynamic range among a range of candidates. To this end, we extended a previously reported ratiometric strategy for robust quantification of promoter activity^30,31^ to T7RNAP-driven gene expression: in our ratiometric reporter plasmids, the T7 promoter controlled expression of *mVenus* YFP^32^ as primary output of T7RNAP activity. Simultaneous monitoring of constitutive *mTurquoise2* CFP^33^ expression from the same plasmid allows correction of the primary output for the overall gene expression capacity of the cell under given environmental conditions.

Comparing different ratiometric reporter plasmids for T7RNAP activity, we found that a variant combining a weak mutant T7 promoter P_T7_(-3G)^37^ with a low copy number origin of replication pSC101 (ref. ^45^) exhibited the highest dynamic range (see Fig. 2a). This observation reflects the well-established fact that high cellular activity of T7RNAP can result in toxicity^46^, likely due to decoupling of T7RNAP-driven transcription and host translation^47–49^ and/or depletion of cellular resources such as nucleotides or amino acids^50^. Indeed, the mutation R632S, thought to reduce the processivity of T7RNAP, has been shown to alleviate toxicity associated with this expression system in *E*. *coli*^39^. In our ratiometric assays, presence of the R632S mutation in T7RNAP increased the maximum induction from the T7 promoter by almost 4-fold (see Fig. 2b). Splitting T7RNAP between residues 179 and 180 (ref. ^25^) is another modification to the enzyme which has been shown to both reduce its processivity^51^ and to alleviate toxicity in *E*. *coli*^26^. In our experiments, (i) introducing the R632S mutation into intact T7RNAP and (ii) splitting the enzyme increased the maximum induction of activity from the T7 promoter to a similar extent. The inducer concentration required for half maximal activity from the T7 promoter was also similarly increased in T7*RNAP and split T7RNAP (~30-fold) compared to T7RNAP (see Supplementary Figure 2). Our results confirm an earlier report of split T7RNAP resolving loss of activity from the T7 promoter upon high enzyme induction^26^.

We also tested variants of split T7RNAP modified by either the R632S mutation, the addition of SynZIP protein–protein interaction domains^40,41^, or both of these features. Interestingly, with split T7RNAP, the individual modifications decreased the maximum induction of activity from the T7 promoter to approximately a third of its original value, with combination of the R632S mutation and SynZIP domains reducing it another 10-fold. The inducer concentration required for half maximal activity was also markedly increased for these variants (over 100-fold compared to T7RNAP; see Supplementary Figure 2). We concluded that splitting T7RNAP sufficiently reduced its processivity to alleviate toxic effects and further modification of this variant unnecessarily compromised its activity.

As the next step towards a synthetic patterning circuit, we sought to build on the proven capacity of split T7RNAP to implement AND-logic^26–28^, and to make this behavior dependent on spatial coincidence of the diffusible signaling molecules 3OC6HSL and 3OC12HSL. In our bicistronic controller plasmid pCRB DRT7VTPLux*500 (see Fig. 3), the N- and C-terminal fragments of split T7RNAP were controlled by promoters induced by either of the two HSLs. Those promoters were derived from our previously reported system for orthogonal intercellular signaling^24^, but modified by mutations shown to reduce basal activity from the Lux promoter by approximately 6-fold^42^. As visual indicators of expression of the individual T7RNAP fragments in vivo, we chose to include the fluorescent reporter genes *mTurquoise2* (downstream of the 3OC6HSL-responsive P_Lux76*_ promoter and *T7RNAP[180-880]*) and *mVenus* (downstream of the 3OC12HSL-responsive P_Las81*_ promoter and *T7RNAP*[1–179]) in the bicistronic controller plasmid. Both were correctly induced by their cognate HSLs in suspension (see Table 1, Supplementary Figure 4). However, a notable level of activity from the T7 promoter as indicated by the RFP reporter plasmid was observed for the bicistronic controller system in the absence of supplemented 3OC12HSL (up to approximately a third of maximum circuit induction). This suggested that the two constitutive half circuits were not perfectly orthogonal, but the promoter P_Las81*_ was induced by 3OC6HSL to some extent. To alleviate this effect, we tested several weak ribosomal binding sites designed by the Ribosome Binding Site Calculator^43^ to control expression of *T7RNAP*[1–179] downstream of P_Las81*_ (see Supplementary Figure 6). We identified a variant RBS_250_ capable of enhancing the maximum circuit induction in the presence of both HSLs to over 8-fold of the maximum background detected in the absence of either input signal (see Fig. 4, Supplementary Figure 8). Interestingly, we observed that corrected fluorescence intensity in both the CFP and the YFP channels was reduced by over 25% under conditions exhibiting high levels of RFP fluorescence compared to conditions inducing either controller half circuit alone. This may be a consequence of rapid transcription of the *mRFP1* gene by T7RNAP relative to *mVenus* and *mTurquoise2* transcribed by endogenous *E*. *coli* RNA polymerase^46–49^, leading to the latter two transgenes being partially out-competed for cellular gene expression capacity. Post-transcriptional limitation of fluorescent protein expression is further suggested by the inverse correlation between CFP expression and RBS strength controlling the YFP operon observed in solid culture assays (Fig. 6). Alternatively, increased expression of certain fluorophores could result in quenching of the others.

Our synthetic three-color patterning circuit also behaved in an AND gate-like manner in surface culture (see Fig. 5), albeit with a reduced dynamic range (see Supplementary Figure 9). The previously mentioned reduction in CFP and YFP fluorescence intensities under conditions of high RFP expression was more pronounced in the surface-based experiment than in suspension culture. One possible explanation for this observation lies in comparatively slow bacterial growth rates during surface culture^52^, which can lead to intracellular accumulation of proteins^53^: in the case of T7RNAP components, protein build-up may promote leaky expression of *mRFP1* from the T7 promoter and increase out-competition of *mVenus* and *mTurquoise2* for cellular gene expression capacity, as discussed above. Intracellular accumulation of fluorescent proteins may also promote quenching due to molecular crowding effects.

Both in suspension (see Fig. 4) and on minimal nutrient agar containing a uniform concentration of HSLs (see Fig. 5a, Supplementary Figure 9), we observed induction of RFP fluorescence to be primarily limited by 3OC12HSL. This is consistent with low-level response of P_Las81*_ to 3OC6HSL. In an earlier report, we have shown fluorescent proteins directly controlled by P_Lux76_ and P_Las81_ to be induced by their cognate homoserine lactones 3OC6HSL and 3OC12HSL in an effectively orthogonal manner^24^. In this work, T7RNAP serves as a potent amplifier of primary promoter activity, magnifying a previously undetectable level of crosstalk.

Despite this effect, our synthetic three-color circuit was capable of generating the emergence of a new pattern of gene expression in response to opposite inducer gradients applied externally (see Fig. 5b) or produced autonomously (see Fig. 6). Under the former condition, we observed a comparatively flat peak in corrected RFP fluorescence across the isogenic bacterial population, likely shaped by the high concentration of externally applied signaling molecules. By contrast, a sharp domain strongly expressing RFP was generated at the interface of 3OC6HSL- and 3OC12HSL-sending cell populations.

**Fig. 7:**
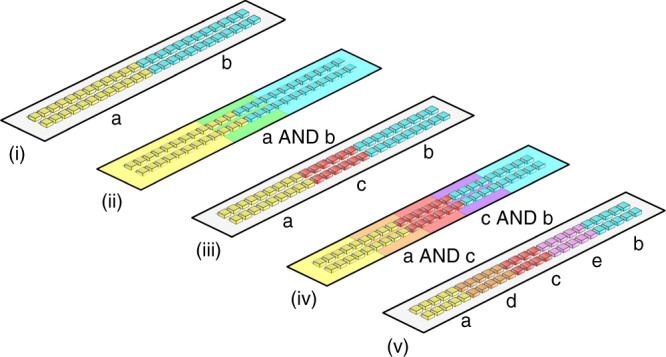
A general framework for hierarchical patterning of bacterial populations. (i) An initial asymmetry splits a bacterial population into two different gene expression domains (yellow boxes and cyan boxes). The yellow domain produces a diffusible morphogen a (yellow background), the cyan domain a diffusible morphogen b (cyan background). (ii) Morphogens a and b coincide at the boundary of the yellow and cyan domains (green background). (iii) Population-based logic induces a red gene expression domain in response to a AND b (red boxes). The red domain produces an additional diffusible morphogen c (red background). (iv) Morphogens a and c coincide at the boundary of the yellow and red domains (orange background), morphogens c and b at the boundary of red and cyan domains (purple background). (v) Population-based logic induces an orange gene expression domain (orange boxes) in response to a AND c, and a purple gene expression domain (purple boxes) in response to c AND b. The orange and purple domains produce additional diffusible morphogens d and e, respectively. This scheme may be iterated to generate (2 × *n*) – 1 different domains with *n* orthogonal signaling systems

This demonstration opens a door to programming hierarchical patterning behavior across populations of cells. For example, replacing the *mRFP1* gene in our synthetic three-color circuit by a biosynthetic enzyme producing a third diffusible signal orthogonal to 3OC6HSL and 3OC12HSL would allow programmed patterning of a multicellular population into five different domains across three hierarchical levels (Fig. 7). Candidate orthogonal signaling systems for this purpose have already been described^22,23,54^. In principle, the population-based AND gate can be iterated with *n* orthogonal signaling systems to generate (2 × *n*) – 1 different domains.

The implementation of higher-order hierarchical patterning processes may become limited by insufficient signal-to-noise ratio in effector expression. In this respect, our approach to implementation of two-layer synthetic hierarchical patterning underlines the importance of empirically characterizing individual genetic components prior to their application in a complex synthetic circuit. As illustrated by the performance of suboptimal bicistronic controller plasmid variants in solid culture assay (see Fig. 6), modulation of a single ribosomal binding site was capable of qualitatively changing circuit behavior: exchange of RBS_250_ for the stronger RBS_B0033_ or the weaker RBS_50_ upstream of *T7RNAP*[1–179] both shifted the balance between CFP and YFP expression in the first layer of patterning and affected the signal-to-noise ratio in RFP expression in the second layer of patterning. Our success in designing a synthetic genetic circuit implementing the desired behavior was dependent on empirical selection of a high-dynamic-range variant of split T7RNAP through ratiometric assays (see Fig. 2) and on translational tuning of the resulting AND gate through plate fluorometer assays (see Supplementary Figure 6).

In nature, morphogen gradients mediate developmental patterning at a high level of precision in face of various perturbations, supported by sophisticated genetic circuits and layers of feedback mechanisms^55–57^. Likewise, the robustness of synthetic hierarchical patterning could be increased by supplementary mechanisms implementing positive feedback or morphogen degradation, for example. In terms of the circuit introduced herein, positive feedback could be implemented by expression of a *T7RNAP* gene under control of the T7 promoter, rendering maintenance of the second layer of patterning independent from the first layer inducers 3OC6HSL and 3OC12HSL. To alleviate potential propagation of signaling crosstalk to higher levels of patterning, the first layer inducers 3OC6HSL and 3OC12HSL could further be specifically degraded at the second layer of patterning. This could be achieved by expression of a quorum-quenching enzyme—such as the lactonase *aiiA*^58^—under control of the T7 promoter. Feedback mechanisms like the ones outlined can be used to consolidate induced domains and to promote correct transmission of spatial information across layers of complexity. As a test-bed for the development of new synthetic feedback circuits, our system for synthetic hierarchical patterning promises to facilitate future efforts at creating custom multicellular organisms for applications in bioproduction, remediation, or medicine.

## Methods

### Plasmid construction

All plasmids (listed in Supplementary Table 4) were constructed using Gibson assembly^59^ with parts obtained from the MIT Registry of Standard Biological Parts (http://partsregistry.org), from Addgene (www.addgene.org), or synthesized by Integrated DNA Technologies (Coralville, IA, USA), and are available on Addgene. Identities and source of backbone vectors, genes, and regulatory elements used in this work are summarized in Supplementary Tables 1–3. Sequences are available on Genbank (accession numbers KY643824 and KX986152 to KX986173). Controller devices encoding intact and split variants of *T7RNAP* were based on plasmids acquired from Shis and Bennett^26^. Bicistronic controller devices were based on a double receiver plasmid previously reported^24^. All cloning was performed in *TOP10 E*. *coli* (Invitrogen, Waltham, MA). With exception of ratiometric reporter plasmids for T7RNAP activity (*T7 Express E*. *coli*; New England Biolabs, Ipswich, MA, USA) all analysis was also carried out in *TOP10 E*. *coli*.

### Plate fluorometer assays

Overnight cultures were diluted 1:100 in M9 medium supplemented with 0.2%_w/v_ casamino acids, 0.4%_w/v_ glucose, and inducers to the concentrations described, and loaded in a final volume of 200 μL per well onto a clear-bottom 96-well microplate (Greiner, Kremsmünster, Austria). Measurements of CFP (excitation 430/10 nm, emission 480/10 nm), YFP (excitation 500/10 nm, emission 530/10 nm), and RFP (excitation 550/10 nm, emission 610/20 nm) fluorescence intensities, and OD_600_ were taken approximately every 12 min for 100 cycles (approximately 19 h) in a BMG FLUOstar Omega plate fluorometer (BMG Labtech, Ortenberg, Germany) at 37 °C, under shaking at 200 rpm between readings. Data analysis was performed using the Genetic Engineering of Cells (GEC) modeling and design environment (version 6.12.2014)^30^.

### Solid culture assays

Single colonies were picked from LB agar plates and grown overnight in supplemented M9 medium with appropriate antibiotics (50 μg/mL carbenicillin and 50 μg/mL kanamycin for double-transformed cells; 50 μg/mL carbenicillin, 50 μg/mL kanamycin, and 25 μg/mL chloramphenicol for triple-transformed cells). Cultures were diluted 1:100, then grown into exponential phase to an optical density at 600 nm of 0.3. This dilute culture was spotted onto Iso-Grid membranes (40 × 40 quadrants of 1 mm per side; Neogen, Lansing, MI, USA) placed on 1.5%_w/v_ agar plates containing the same supplemented M9 growth medium. The culture was plated at a volume of 0.5 μL per quadrant. Plates were incubated and imaged in a custom imaging device which has been described in detail elsewhere^24^. For quantification of fluorescence intensities, mean pixel gray values localized to individual Iso-Grid quadrants across the CFP, YFP, and RFP channels were extrapolated using the open-source Fiji distribution of ImageJ^60^.

### Data availability

The sequences of 23 plasmids used in this study have been submitted to the GenBank nucleotide database under accession codes KY643824 and KX986152 to KX986173. The authors declare that all data supporting the findings of this study are available within the paper and its Supplementary Information files or are available from the corresponding author on request.

## Electronic supplementary material

Supplementary Information
