# Supplementary material for: Programmed hierarchical patterning of bacterial populations
Source: Nat Commun. 2018 Feb 22;9:776. doi: 10.1038/s41467-018-03069-3 (PMC5823926; doi:10.1038/s41467-018-03069-3)
Supplement: Supplementary file 1 — Supplementary Information [file 41467_2018_3069_MOESM1_ESM.pdf]

Supplementary Information

Programmed hierarchical patterning of bacterial populations

Boehm et al.

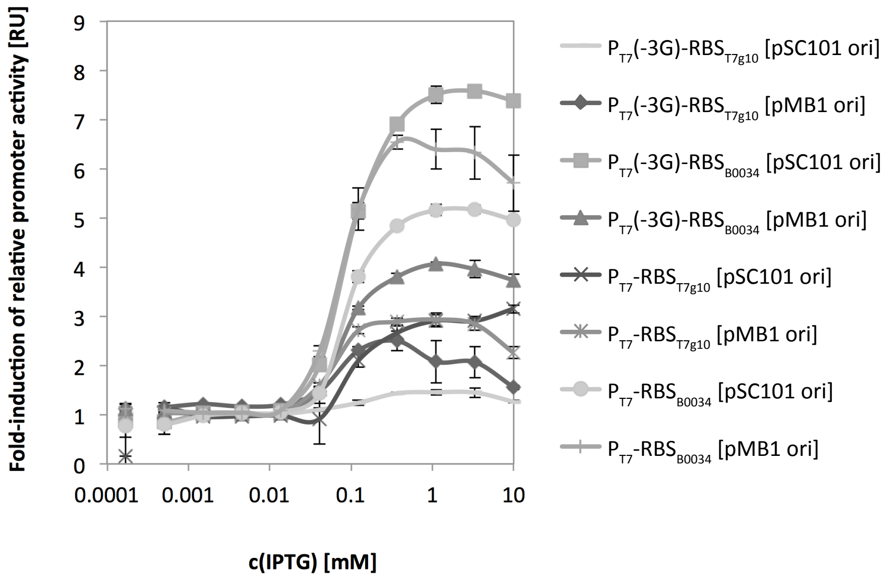

Supplementary Figure 1 | Induction curves of ratiometric reporter plasmids. Ratiometric reporter plasmids combining different variants of the T7 promoter, 5'-UTRs, and origins of replication were introduced into *T7 Express E. coli* (see Figure 2a). The fold-induction of relative activity from the T7 promoter in response to a range of IPTG concentrations was quantified using a plate fluorometer-based assay (see Methods for details), and is reported relative to the absence of inducer. Error bars represent the s.d. of average values yielded between 3 biological replicate experiments performed on different days.

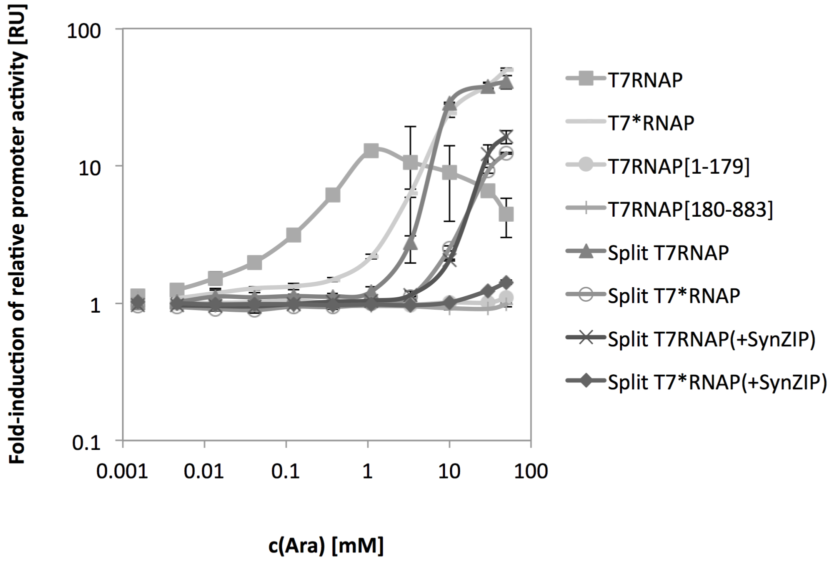

Supplementary Figure 2 | Induction curves of intact and split *T7RNAP* variants. *TOP10 E. coli* were co-transformed by the ratiometric reporter plasmid p4g3VT (see 'P_{T7}(-3G)-RBS_{B0034} [pSC101 ori]' in Supplementary Figure 1) and different controller plasmids encoding intact or fragmented variants of the *T7RNAP* gene under control of the arabinose-inducible $P_{BAD/araC}$ promoter (see Figure 2b). The fold-induction of relative activity from the T7 promoter in response to a range of arabinose concentrations was quantified using a plate fluorometer-based assay (see Methods for details), and is reported relative to the absence of inducer. Error bars represent the s.d. of average values yielded between 3 biological replicate experiments performed on different days.

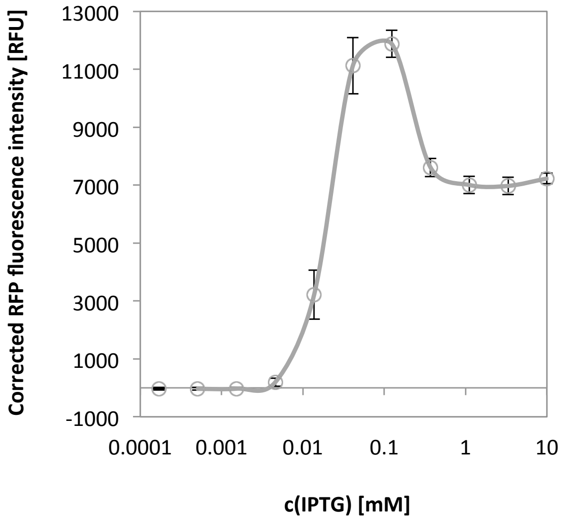

Supplementary Figure 3 | Induction curve of the RFP reporter plasmid p4g3R. The RFP reporter plasmid p4g3R was introduced into *T7 Express E. coli*. RFP fluorescence intensity was quantified as a function of IPTG using a plate fluorometer-based assay (see Methods for details), and corrected for background signal present in absence of externally supplied inducer. Error bars represent the s.d. of average values yielded between 3 biological replicate experiments performed on different days.

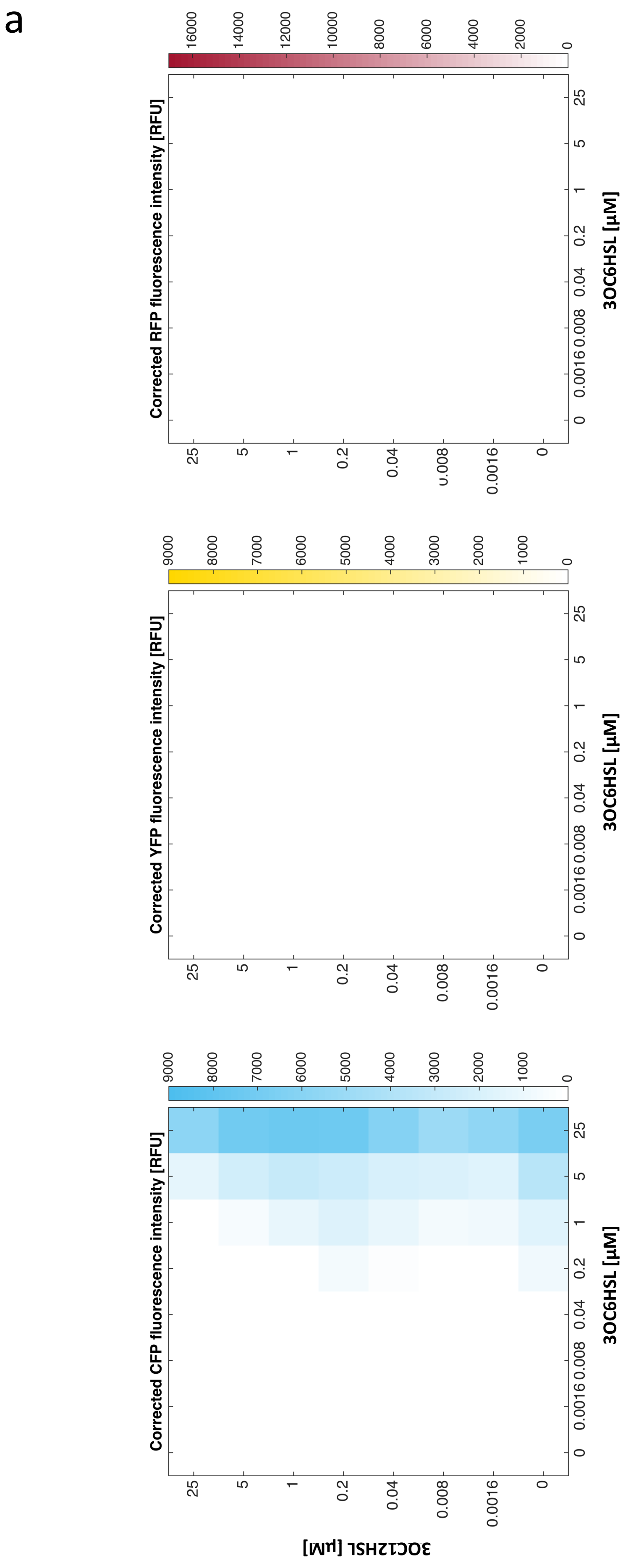
a
Corrected CFP fluorescence intensity [RFU]
Corrected YFP fluorescence intensity [RFU]
Corrected RFP fluorescence intensity [RFU]
3OC6HSL [µM]
3OC12HSL [µM]
0
0.0016
0.008
0.04
0.2
1
5
25
0
1000
2000
3000
4000
5000
6000
7000
8000
9000
0
2000
4000
6000
8000
10000
12000
14000
16000

b

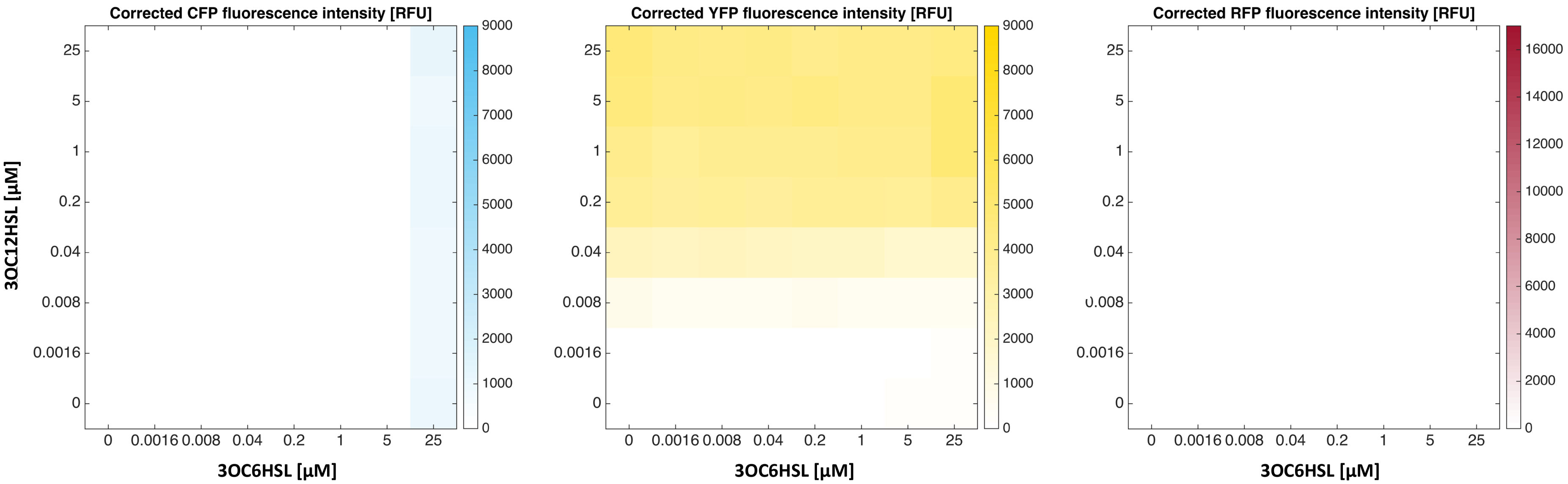
Corrected CFP fluorescence intensity [RFU]
Corrected YFP fluorescence intensity [RFU]
Corrected RFP fluorescence intensity [RFU]
3OC12HSL [μM]
3OC6HSL [μM]
25
5
1
0.2
0.04
0.008
0.0016
0
9000
8000
7000
6000
5000
4000
3000
2000
1000
16000
14000
12000
10000

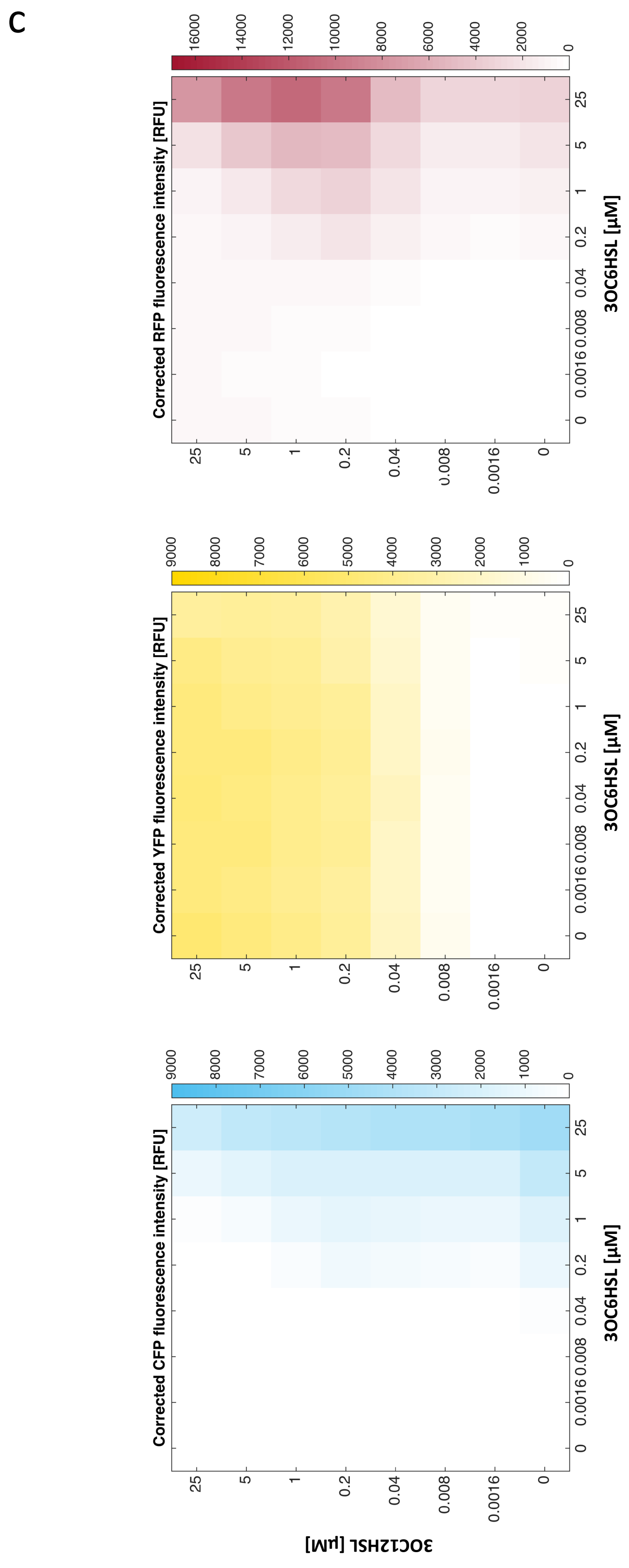
c
Corrected CFP fluorescence intensity [RFU]
Corrected YFP fluorescence intensity [RFU]
Corrected RFP fluorescence intensity [RFU]
3OC6HSL [µM]
3OC12HSL [µM]
0
0.0016
0.008
0.04
0.2
1
5
25

Supplementary Figure 4 | Activity plots of split T7RNAP-based three-color half circuits. *TOP10 E. coli* were co-transformed by an *mRFP1*-expressing reporter of T7RNAP activity p4g3R (see Supplementary Figure 3) and (**a**) half-circuit bicistronic controller plasmid pCRB DRT7VTPLux*500CFP, (**b**) half-circuit bicistronic controller plasmid pCRB DRT7VTPLux*500YFP, or (**c**) bicistronic controller plasmid pCRB DRT7VTPLux*500. Compared to pCRB DRT7VTPLux*500, pCRB DRT7VTPLux*500CFP and pCRB DRT7VTPLux*500YFP lacked the bicistronic operon P_{Las81*}-*T7RNAP[1-179]-mVenus* or P_{Lux76*}-*T7RNAP[180-880]-mTurquoise2*, respectively. The behavior of bicistronic controller plasmids was tested alongside the RFP reporter plasmid under a two-dimensional titration of 3OC6HSL and 3OC12HSL using a plate fluorometer-based assay (see Methods for details). Fluorescence intensity, corrected for background signal present in absence of externally supplied homoserine lactones, is reported for each condition. Plots show average values from 3 biological replicate experiments performed on different days. Corresponding s.d. values are shown alongside individual induction curves in Supplementary Figure 5.

a

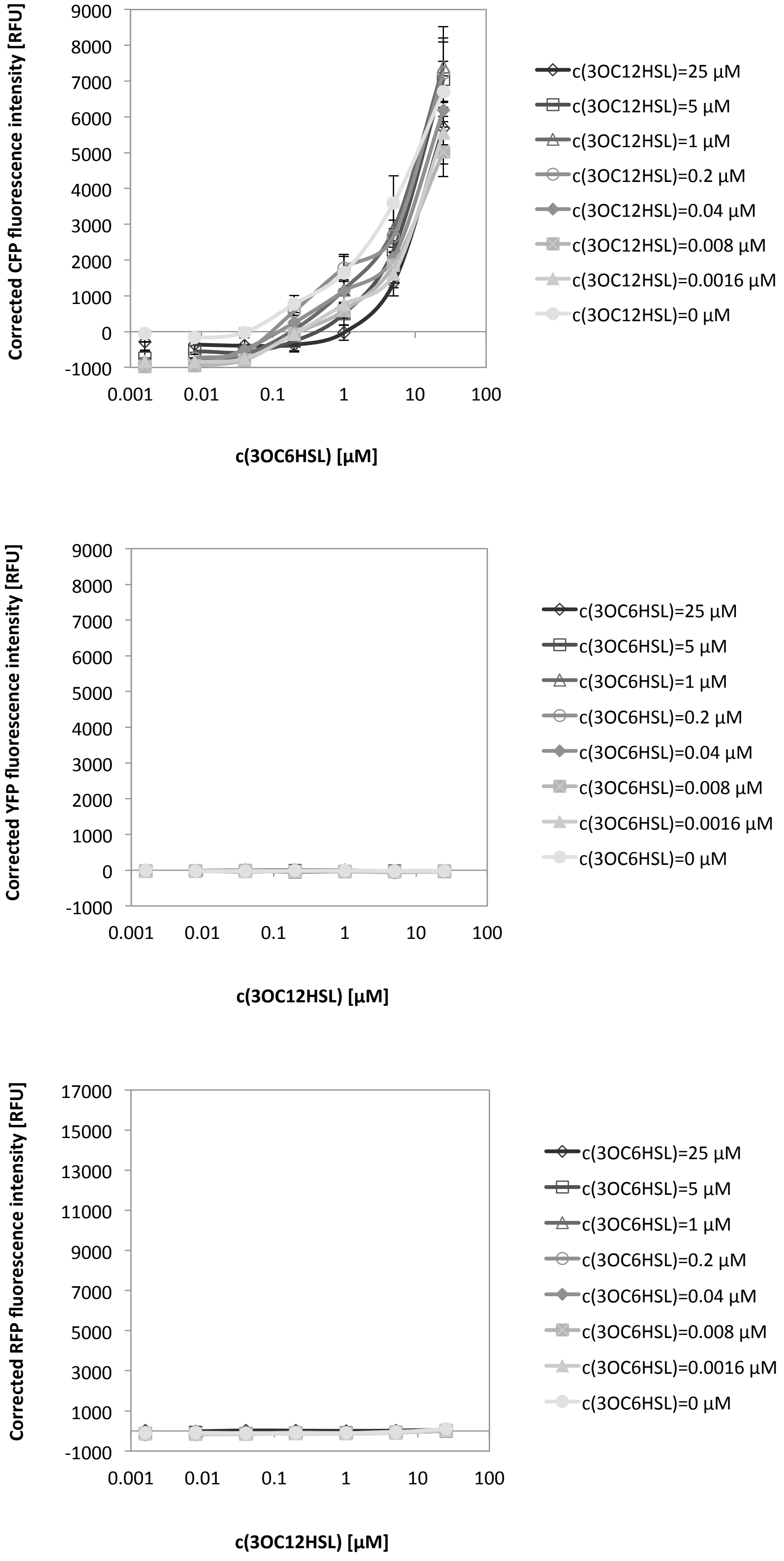
Corrected CFP fluorescence intensity [RFU]
9000
8000
7000
6000
5000
4000
3000
2000
1000
0
-1000
0.001
0.01
0.1
1
10
100
c(3OC6HSL) [µM]
c(3OC12HSL)=25 µM
c(3OC12HSL)=5 µM
c(3OC12HSL)=1 µM
c(3OC12HSL)=0.2 µM
c(3OC12HSL)=0.04 µM
c(3OC12HSL)=0.008 µM
c(3OC12HSL)=0.0016 µM
c(3OC12HSL)=0 µM
Corrected YFP fluorescence intensity [RFU]
9000
8000
7000
6000
5000
4000
3000
2000
1000
0
-1000
0.001
0.01
0.1
1
10
100
c(3OC12HSL) [µM]
c(3OC6HSL)=25 µM
c(3OC6HSL)=5 µM
c(3OC6HSL)=1 µM
c(3OC6HSL)=0.2 µM
c(3OC6HSL)=0.04 µM
c(3OC6HSL)=0.008 µM
c(3OC6HSL)=0.0016 µM
c(3OC6HSL)=0 µM
Corrected RFP fluorescence intensity [RFU]
17000
15000
13000
11000
9000
7000
5000
3000
1000
-1000
0.001
0.01
0.1
1
10
100
c(3OC12HSL) [µM]
c(3OC6HSL)=25 µM
c(3OC6HSL)=5 µM
c(3OC6HSL)=1 µM
c(3OC6HSL)=0.2 µM
c(3OC6HSL)=0.04 µM
c(3OC6HSL)=0.008 µM
c(3OC6HSL)=0.0016 µM
c(3OC6HSL)=0 µM

b

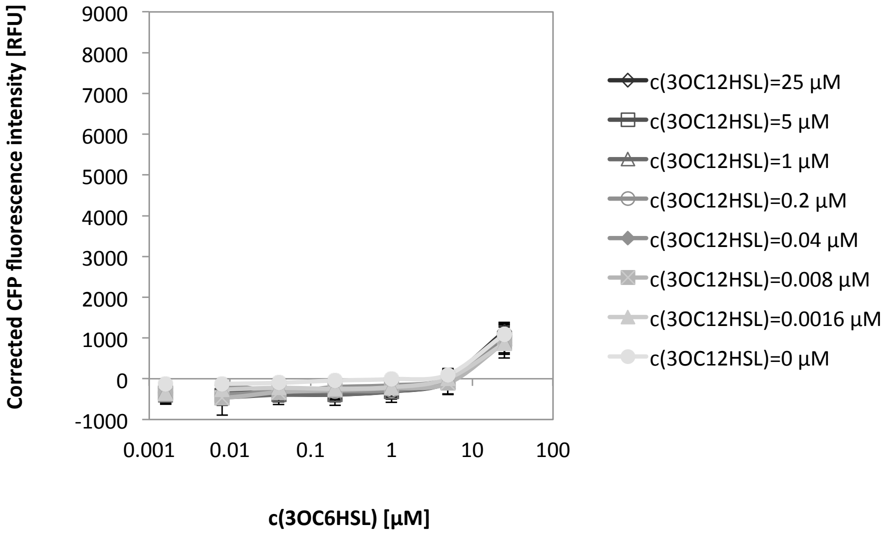
Corrected CFP fluorescence intensity [RFU]
9000
8000
7000
6000
5000
4000
3000
2000
1000
0
-1000
0.001
0.01
0.1
1
10
100
c(3OC6HSL) [µM]
c(3OC12HSL)=25 µM
c(3OC12HSL)=5 µM
c(3OC12HSL)=1 µM
c(3OC12HSL)=0.2 µM
c(3OC12HSL)=0.04 µM
c(3OC12HSL)=0.008 µM
c(3OC12HSL)=0.0016 µM
c(3OC12HSL)=0 µM

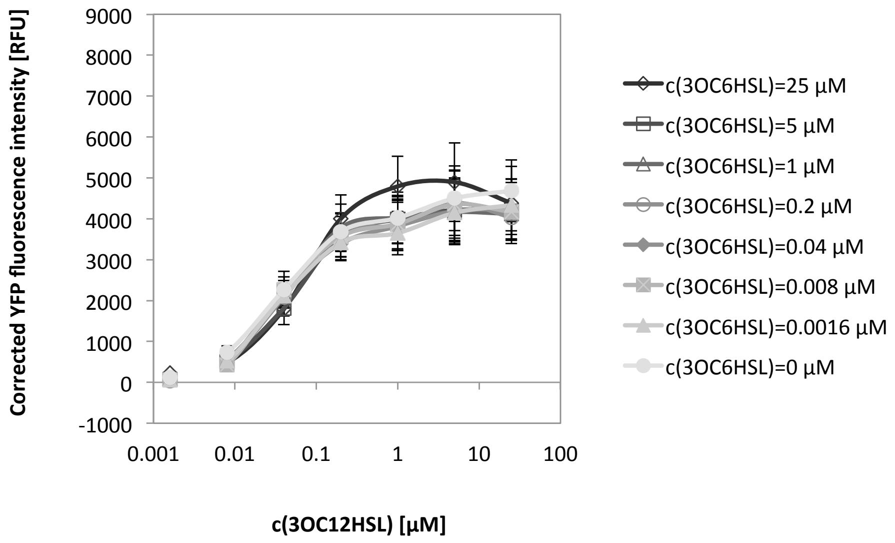
Corrected YFP fluorescence intensity [RFU]
9000
8000
7000
6000
5000
4000
3000
2000
1000
0
-1000
0.001
0.01
0.1
1
10
100
c(3OC12HSL) [µM]
c(3OC6HSL)=25 µM
c(3OC6HSL)=5 µM
c(3OC6HSL)=1 µM
c(3OC6HSL)=0.2 µM
c(3OC6HSL)=0.04 µM
c(3OC6HSL)=0.008 µM
c(3OC6HSL)=0.0016 µM
c(3OC6HSL)=0 µM

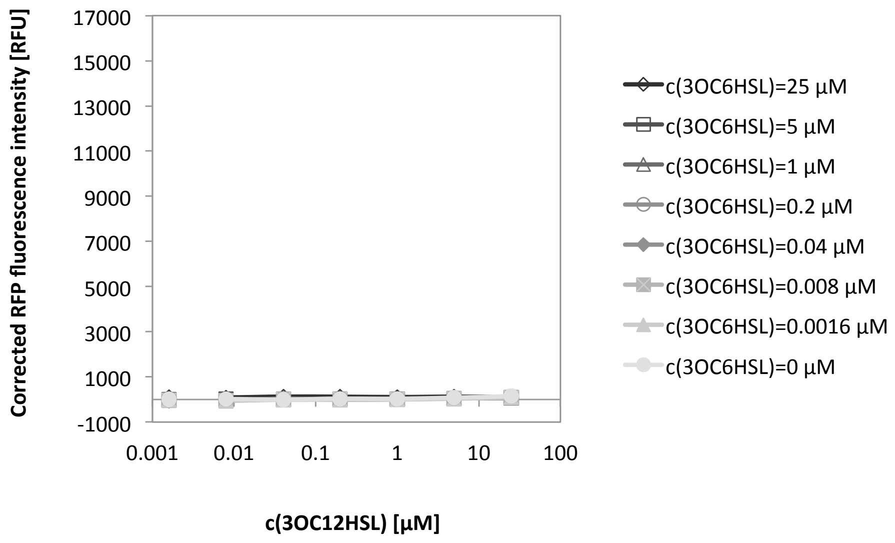
Corrected RFP fluorescence intensity [RFU]
17000
15000
13000
11000
9000
7000
5000
3000
1000
-1000
0.001
0.01
0.1
1
10
100
c(3OC12HSL) [µM]
c(3OC6HSL)=25 µM
c(3OC6HSL)=5 µM
c(3OC6HSL)=1 µM
c(3OC6HSL)=0.2 µM
c(3OC6HSL)=0.04 µM
c(3OC6HSL)=0.008 µM
c(3OC6HSL)=0.0016 µM
c(3OC6HSL)=0 µM

C

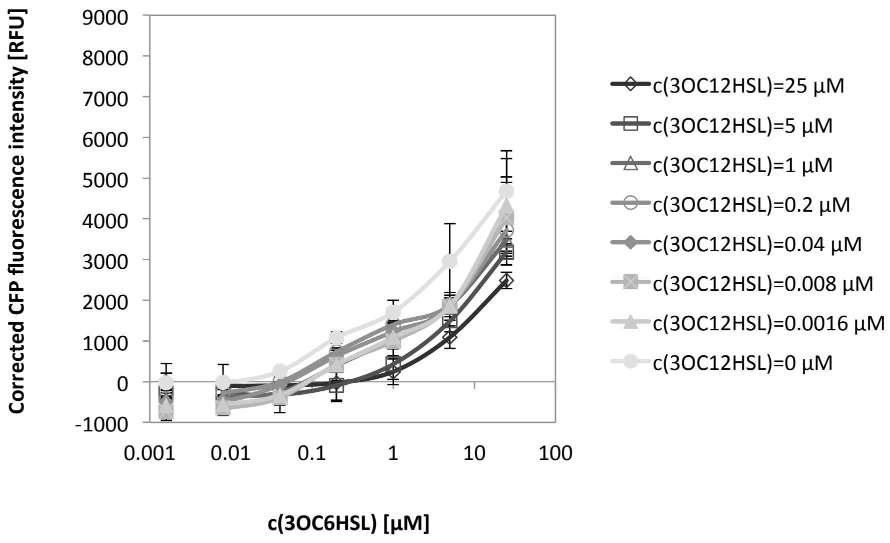
Corrected CFP fluorescence intensity [RFU]
9000
8000
7000
6000
5000
4000
3000
2000
1000
0
-1000
0.001
0.01
0.1
1
10
100
c(3OC6HSL) [µM]
c(3OC12HSL)=25 µM
c(3OC12HSL)=5 µM
c(3OC12HSL)=1 µM
c(3OC12HSL)=0.2 µM
c(3OC12HSL)=0.04 µM
c(3OC12HSL)=0.008 µM
c(3OC12HSL)=0.0016 µM
c(3OC12HSL)=0 µM

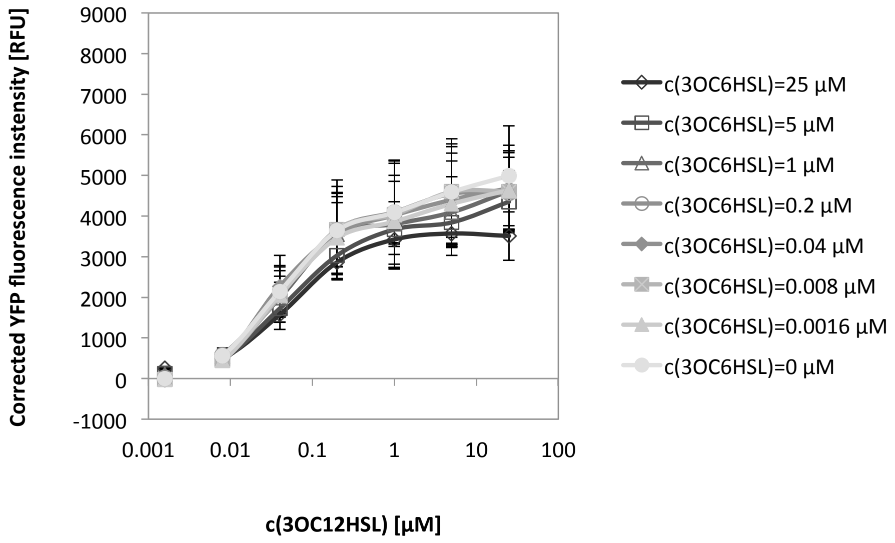
Corrected YFP fluorescence instensity [RFU]
9000
8000
7000
6000
5000
4000
3000
2000
1000
0
-1000
0.001
0.01
0.1
1
10
100
c(3OC12HSL) [µM]
c(3OC6HSL)=25 µM
c(3OC6HSL)=5 µM
c(3OC6HSL)=1 µM
c(3OC6HSL)=0.2 µM
c(3OC6HSL)=0.04 µM
c(3OC6HSL)=0.008 µM
c(3OC6HSL)=0.0016 µM
c(3OC6HSL)=0 µM

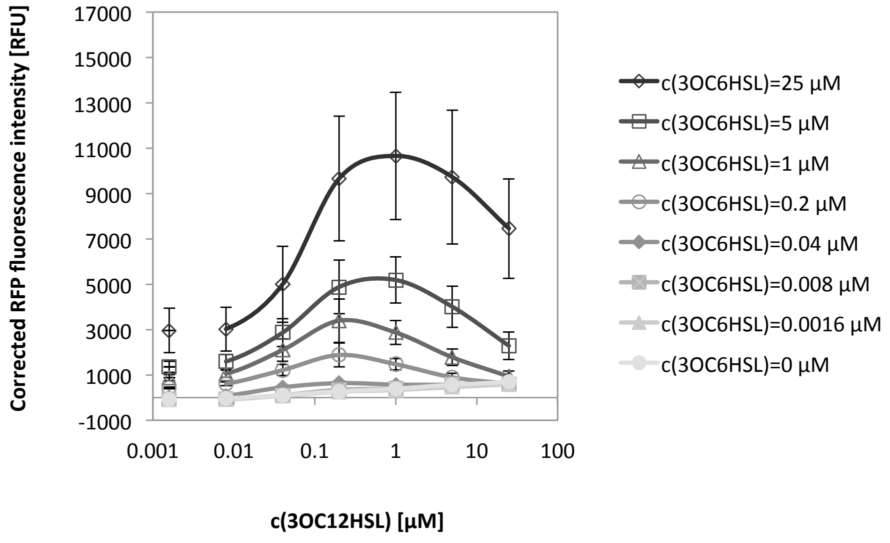
Corrected RFP fluorescence intensity [RFU]
17000
15000
13000
11000
9000
7000
5000
3000
1000
-1000
0.001
0.01
0.1
1
10
100
c(3OC12HSL) [µM]
c(3OC6HSL)=25 µM
c(3OC6HSL)=5 µM
c(3OC6HSL)=1 µM
c(3OC6HSL)=0.2 µM
c(3OC6HSL)=0.04 µM
c(3OC6HSL)=0.008 µM
c(3OC6HSL)=0.0016 µM
c(3OC6HSL)=0 µM

Supplementary Figure 5 | Induction curves of a split T7RNAP-based three-color circuit. *TOP10 E. coli* were co-transformed by the RFP reporter plasmid of T7RNAP activity p4g3R (see Supplementary Figure 3) and (**a)** half-circuit bicistronic controller plasmid pCRB DRT7VTPLux*500CFP, (**b**) half-circuit bicistronic controller plasmid pCRB DRT7VTPLux*500YFP, or (**c**) bicistronic controller plasmid pCRB DRT7VTPLux*500. pCRB DRT7VTPLux*500 encoded two bicistronic operons responding to 3OC6HSL and 3OC12HSL by induction of *T7RNAP[180-880]* and *mTurquoise2*, or *T7RNAP[1-179]* and *mVenus*, respectively. pCRB DRT7VTPLux*500CFP and pCRB DRT7VTPLux*500YFP lacked the bicistronic operon P_{Las81*}-*T7RNAP[1-179]-mVenus* or P_{Lux76*}-*T7RNAP[180-880]-mTurquoise2*, respectively. The behavior of half-circuit bicistronic controller plasmids (see (a) and (b)) and the bicistronic controller plasmid (see (c)) was tested alongside the RFP reporter plasmid under a two-dimensional titration of 3OC6HSL and 3OC12HSL using a plate fluorometer-based assay (see Methods for details). Fluorescence intensity, corrected for background signal present in absence of externally supplied homoserine lactones, is reported for each condition. Error bars represent the s.d. of average values yielded between 3 biological replicate experiments performed on different days. Activity plots are shown in Supplementary Figure 4.

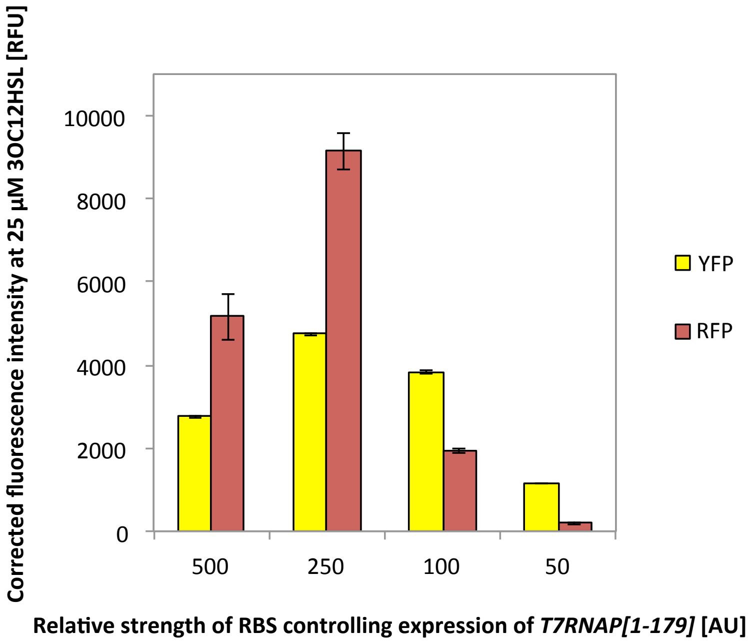

Supplementary Figure 6 | Comparison of split T7RNAP-based three-color circuit variants of varying RBS strength controlling *T7RNAP[1-179]*. *TOP10 E. coli* co-transformed by the RFP reporter plasmid p4g3R and bicistronic controller plasmid variants pCRB DRT7VTPLux*(500/250/100/50) were subjected to a plate fluorometer-based assay (see Methods for details) in presence of 25 µM 3OC6HSL. Fluorescence intensity, corrected for background signal present in absence of 3OC12HSL, is reported (see Supplementary Figure 7 for individual induction curves). Error bars represent the s.d. of average values between 3 biological replicate experiments starting from different colonies.

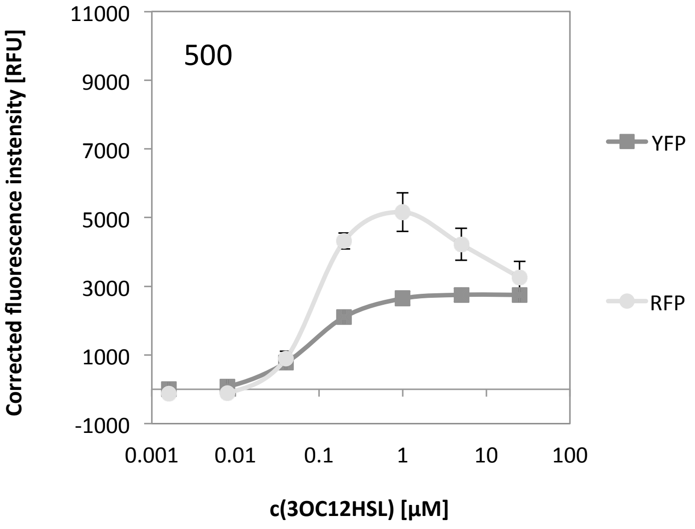
500
11000
9000
7000
5000
3000
1000
-1000
0.001
0.01
0.1
1
10
100
Corrected fluorescence instensity [RFU]
c(3OC12HSL) [µM]
YFP
RFP

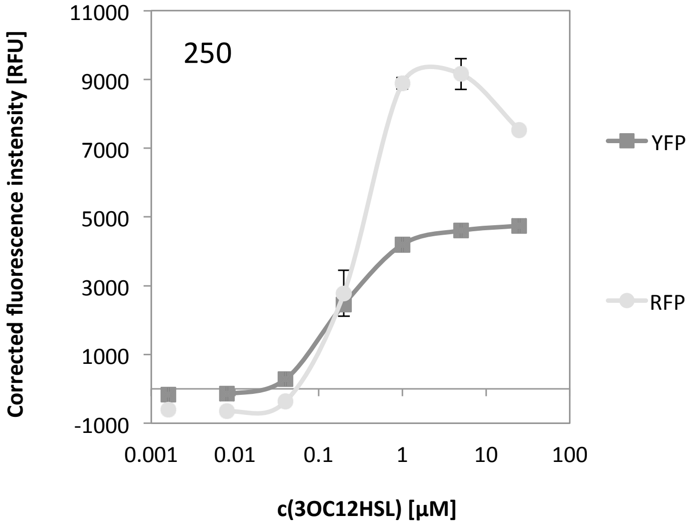
250
11000
9000
7000
5000
3000
1000
-1000
0.001
0.01
0.1
1
10
100
Corrected fluorescence instensity [RFU]
c(3OC12HSL) [µM]
YFP
RFP

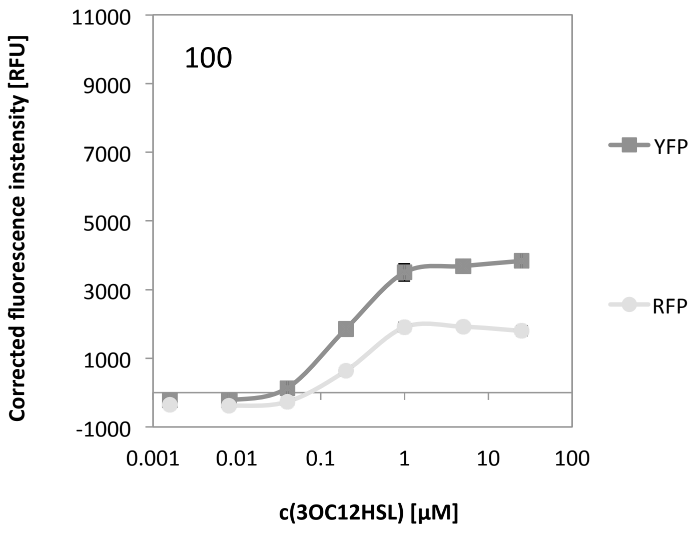

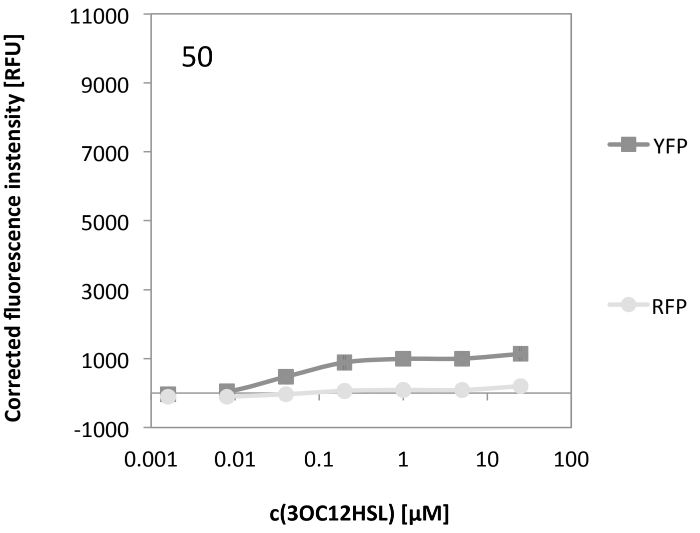

Supplementary Figure 7 | Induction curves of split T7RNAP-based three-color circuit variants of varying RBS strength controlling *T7RNAP[1-179]*. *TOP10 E. coli* were co-transformed by the RFP reporter plasmid p4g3R (see Supplementary Figure 3) and different variants of the bicistronic controller plasmid pCRB DRT7VTPLux*(500/250/100/50) featuring ribosomal binding sites of different strength controlling expression of *T7RNAP[1-179]* (see Supplementary Figure 6). The relative strength of the respective RBS according to the Ribosome Binding Site Calculator[1] is shown on the top left corner in each plot. Plate fluorometer-based assays (see Methods for details) were performed in presence of 25 μM 3OC6HSL. Fluorescence intensity, corrected for background signal present in absence of 3OC12HSL, is reported. Error bars represent the s.d. of average values between 3 biological replicate experiments starting from different colonies.

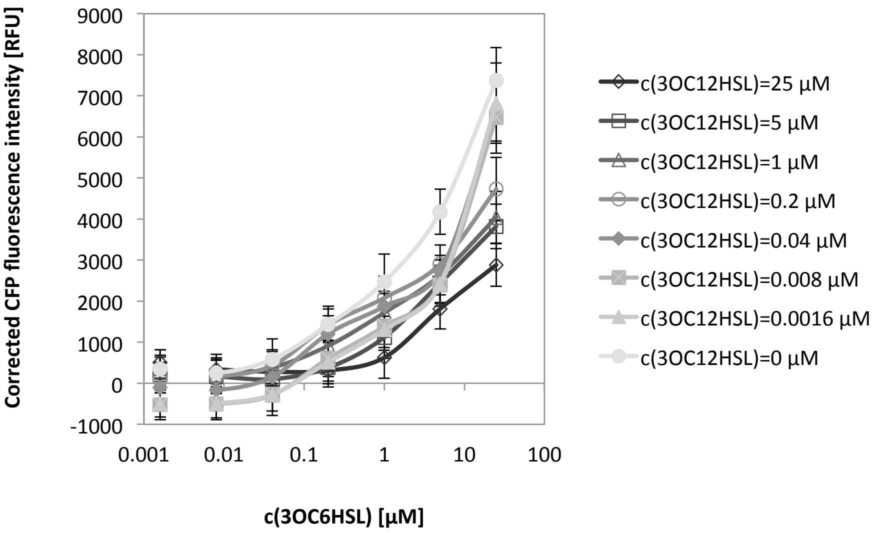
Corrected CFP fluorescence intensity [RFU]
9000
8000
7000
6000
5000
4000
3000
2000
1000
0
-1000
0.001
0.01
0.1
1
10
100
c(3OC6HSL) [µM]
c(3OC12HSL)=25 µM
c(3OC12HSL)=5 µM
c(3OC12HSL)=1 µM
c(3OC12HSL)=0.2 µM
c(3OC12HSL)=0.04 µM
c(3OC12HSL)=0.008 µM
c(3OC12HSL)=0.0016 µM
c(3OC12HSL)=0 µM

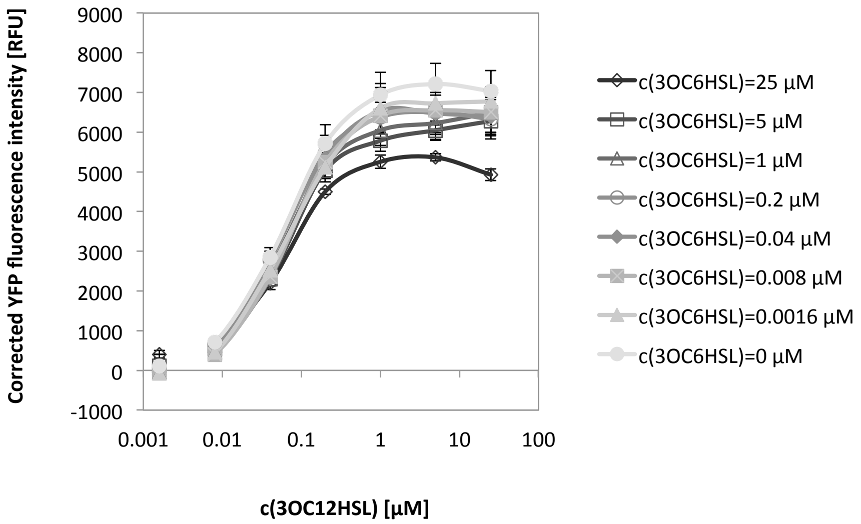
Corrected YFP fluorescence intensity [RFU]
9000
8000
7000
6000
5000
4000
3000
2000
1000
0
-1000
0.001
0.01
0.1
1
10
100
c(3OC12HSL) [µM]
c(3OC6HSL)=25 µM
c(3OC6HSL)=5 µM
c(3OC6HSL)=1 µM
c(3OC6HSL)=0.2 µM
c(3OC6HSL)=0.04 µM
c(3OC6HSL)=0.008 µM
c(3OC6HSL)=0.0016 µM
c(3OC6HSL)=0 µM

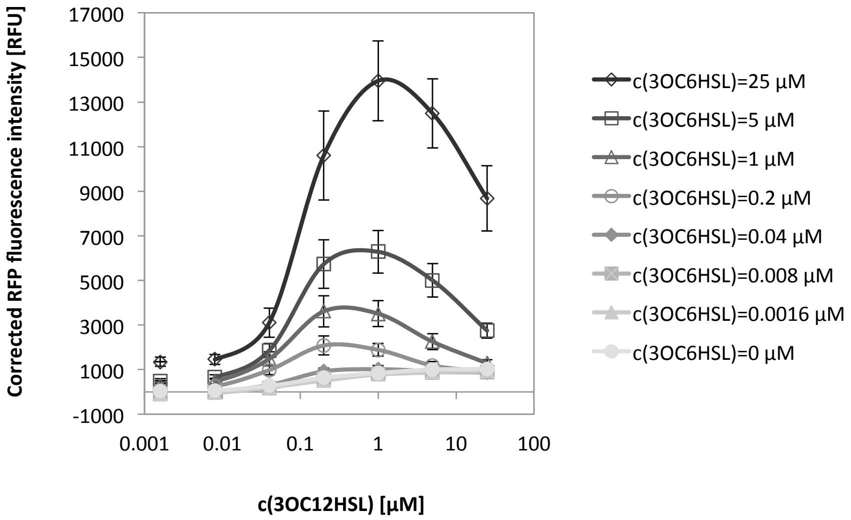
Corrected RFP fluorescence intensity [RFU]
17000
15000
13000
11000
9000
7000
5000
3000
1000
-1000
0.001
0.01
0.1
1
10
100
c(3OC12HSL) [µM]
c(3OC6HSL)=25 µM
c(3OC6HSL)=5 µM
c(3OC6HSL)=1 µM
c(3OC6HSL)=0.2 µM
c(3OC6HSL)=0.04 µM
c(3OC6HSL)=0.008 µM
c(3OC6HSL)=0.0016 µM
c(3OC6HSL)=0 µM

Supplementary Figure 8 | Induction curves of an improved split T7RNAP-based three-color AND gate. *TOP10 E. coli* were co-transformed by the RFP reporter plasmid of T7RNAP activity p4g3R (see Supplementary Figure 3) and the bicistronic controller plasmid pCRB DRT7VTPLux*250 (see Figure 4, Supplementary Figure 6). The behavior of the bicistronic controller plasmid was tested alongside the RFP reporter plasmid under a two-dimensional titration of 3OC6HSL and 3OC12HSL using a plate fluorometer-based assay (see Methods for details). Fluorescence intensity, corrected for background signal present in absence of externally supplied homoserine lactones, is reported for each condition. Error bars represent the s.d. of average values yielded between 3 biological replicate experiments performed on different days.

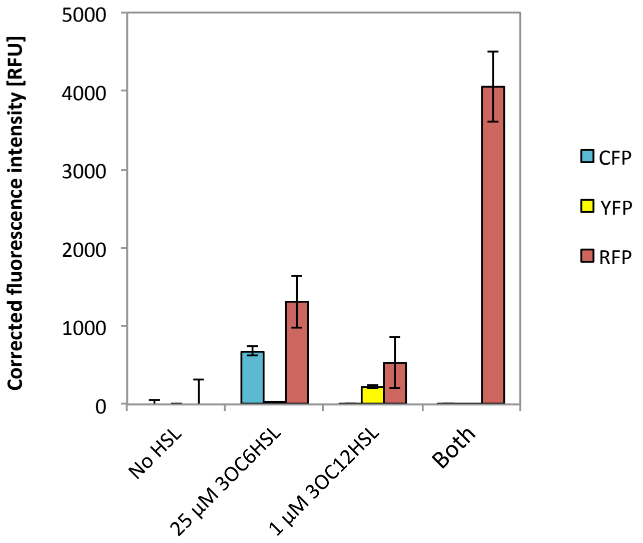

Supplementary Figure 9 | Surface-based behavior of a split T7RNAP-based three-color AND gate in response to homoserine lactones at uniform concentration. *TOP10 E. coli* co-transformed by the RFP reporter plasmid p4g3R and the improved bicistronic controller plasmid pCRB DRT7VTPLux*250 (see Figure 4) were incubated on membranes printed with hydrophobic ink, placed on minimal agar containing different combinations of homoserine lactones (see Figure 5a). Fluorescence intensity at t = 1,500 min (time relative to start of incubation), corrected for background signal present in absence of externally supplied homoserine lactones, is reported for each condition. Error bars represent the s.d. of average values between the 64 quadrants on each membrane.

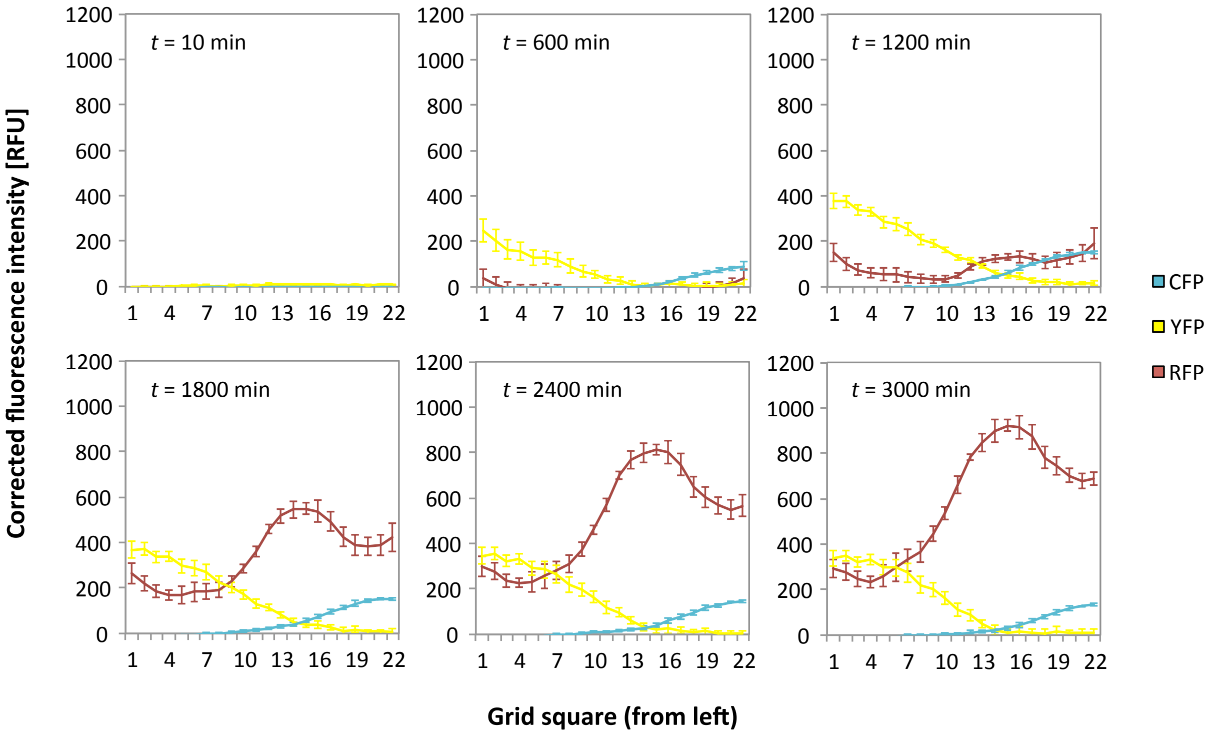

Supplementary Figure 10 | Surface-based behavior of a split T7RNAP-based three-color AND gate in response to homoserine lactone gradients. *TOP10 E. coli* co-transformed by the RFP reporter plasmid p4g3R and the improved bicistronic controller plasmid pCRB DRT7VTPLux*250 (see Figure 4) were incubated on membranes printed with hydrophobic ink, placed on minimal agar lacking supplemented homoserine lactones (see Figure 5b). Instead, aqueous solutions containing 500 μM 3OC6HSL or 200 μM 3OC12HSL, respectively, were spotted next to the cells on either side and left to diffuse into the bacterial population from opposite directions. 3OC6HSL was spotted next to grid square 22, 3OC12HSL next to grid square 1. Fluorescence intensity, corrected for background signal present in absence of externally supplied homoserine lactones, is reported for each set of quadrants equidistant from the position of spotting (4 each). Error bars represent the s.d. of average values between the 4 equidistant quadrants on each membrane.

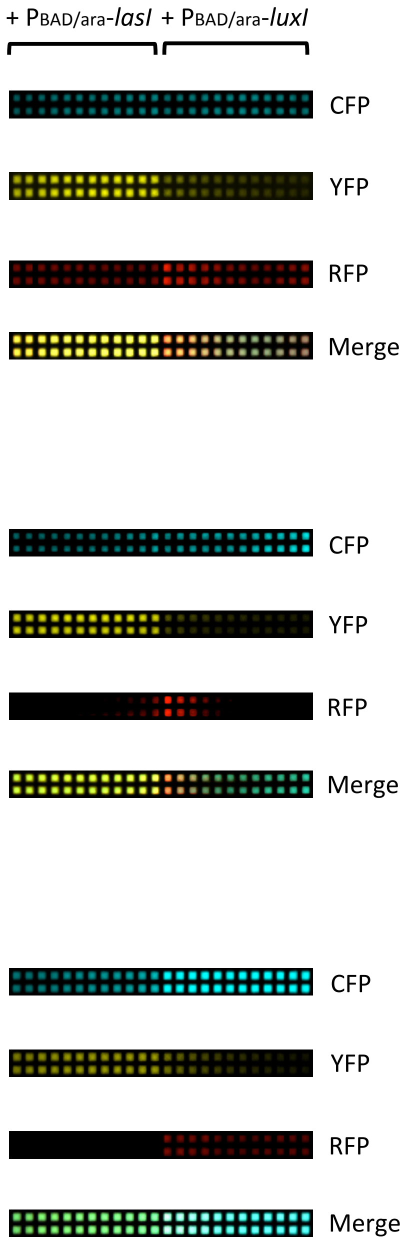

Supplementary Figure 11 | Patterning of bacterial populations in the absence of externally applied homoserine lactones. *TOP10 E. coli* were co-transformed by (i) the RFP reporter plasmid p4g3R, (ii) a bicistronic controller plasmid pCRB DRT7VTPLux*500 (top panel), pCRB DRT7VTPLux*250 (mid panel), or pCRB DRT7VTPLux*50 (bottom panel), and (iii) a sender plasmid pSB1C3 I0500 (LuxI/LasI) encoding either *luxI* or *lasI* under control of the arabinose-inducible $P_{BAD/araC}$ promoter. Adjacent populations of the different triple-transformed cell types were incubated on membranes placed on minimal nutrient agar which has supplemented with 25 mM arabinose (see Figure 6). Images shown were captured at t = 3,000 min.

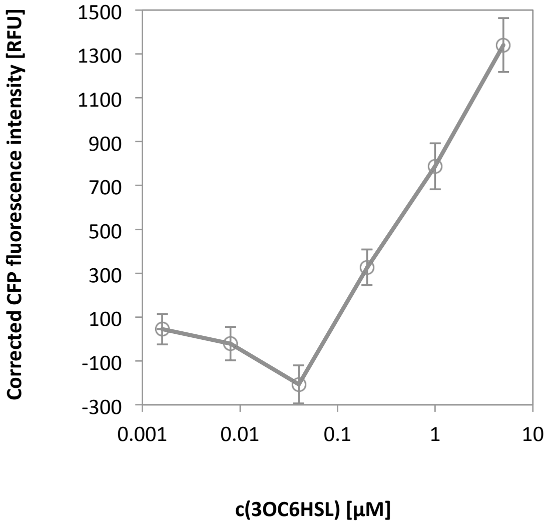

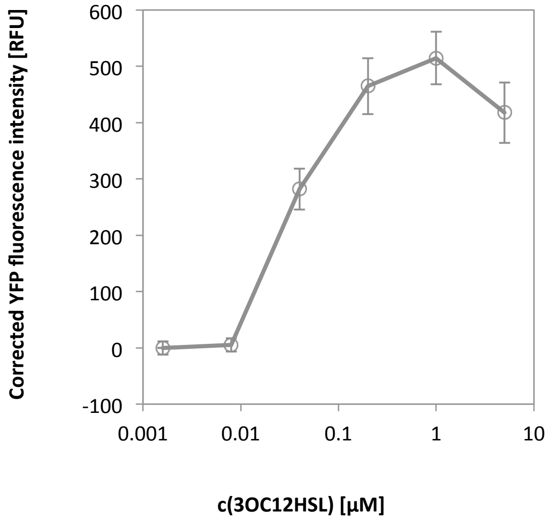

Supplementary Figure 12 | Standard curves for solid culture assays. *TOP10 E. coli* co-transformed by the RFP reporter plasmid p4g3R and the improved bicistronic controller plasmid pCRB DRT7VTPLux*250 (see Figure 4) were incubated on membranes (64 quadrants) printed with hydrophobic ink, placed on minimal agar containing different concentrations of either 3OC6HSL (top) or 3OC12HSL (bottom). Fluorescence intensity, corrected for background signal present in absence of externally supplied homoserine lactones, is reported for each condition. Data shown was collected at t = 3,000 min (time relative to start of incubation). Error bars represent the s.d. of average values between the 64 quadrants on each membrane.

Backbone Name	Source
pSB1A3	Parts Registry
pSB4A5	Parts Registry
pBAD33	Rosenberg *et al.* [2]
pR33S175	Grant *et al.* [3]
pSB1C3	Parts Registry

Supplementary Table 1 | Vector backbones used in this work.

Gene Name	Source
mVenus	pUNVe (Federici *et al.*)[4]
lacI(LVA)	Parts Registry: BBa_P0412
mTurquoise2	pRSET mTurquoise2 (Goedhart *et al.*)[5]
araC	pTara:500 (Shis *et al.*)[6]
T7RNAP	pTara:500 (Shis *et al.*)[6]
mRFP1	Parts Registry: BBa_E1010
luxR	pR33S175 (Grant *et al.*)[3]
lasR	pR33S175 (Grant *et al.*)[3]
luxI	pLas81-LuxI (Grant *et al.*)[3]
lasI	pLux76-LasI (Grant *et al.*)[3]

Supplementary Table 2 | Genes used in this work.

Regulatory Element Name	Regulatory Element Sequence (5'->3')	Source
P_{T7}	TAATACGACTCACTATAGGGAATACAAGCTACTTGTTCTTTTTGCA	Parts Registry: BBa_I712074
P_{T7}(-3G)	TAATACGACTCACTGTAGGGAATACAAGCTACTTGTTCTTTTTGCA	This work
P_{T7}-RBS_{T7g10}	GCGAAATTAATACGACTCACTATAGGGAGACCACAACGGTTTCCC- -TCTAGAAATAATTTTGTTTAACTTTAAGAAGGAGATATAC	pET3a (Rosenberg *et al.*)[2]
P_{T7}(-3G)-RBS_{T7g10}	GCGAAATTAATACGACTCACTGTAGGGAGACCACAACGGTTTCCC- -TCTAGAAATAATTTTGTTTAACTTTAAGAAGGAGATATAC	This work
P_{J23101}	TTTACAGCTAGCTCAGTCCTAGGTATTATGCTAGC	Parts Registry: BBa_J23101
$P_{J23101*}$	TTTACAGCTAGCTCAGTCCTAGGTTTTATGCTAGC	This work
P_{araC}	ATAGCAAAGTGTGACGCCGTGCAAATAATCAATGTGGACTTTTCT- -GCCGTGATTATAGACACTTTTGTTACG	pTara:500 (Shis *et al.*)[6]
P_{BAD}	ACATTGATTATTTGCACGGCGTCACACTTTGCTATGCCATAGCATTTTTATCCATAAGATTAGCGG- -ATCCTACCTGACGCTTTTTATCGCAACTCTCTACTGTTTCTCCATACCCGTTTTTTTGGGCTAGC	pTara:500 (Shis *et al.*)[6]
P_{R0040}	TCCCTATCAGTGATAGAGATTGACATCCCTATCAGTGATAGAGATACTGAGCAC	pR33S175 (Grant *et al.*)[3]
P_{R0011}	AATTGTGAGCGGATAACAATTGACATTGTGAGCGGATAACAAGATACTGAGCACA	pR33S175 (Grant *et al.*)[3]
P_{Lux76*}	ACCTGTAGGATCGTACAAGTTTACGCAAGAAAATGGTTTGTTACTTTCGAATAAA	This work
P_{Las81*}	ACCTATAGGATCGTATAGGTTTACGCAAGAAAATGGTTTGTTACTTTCGAATAAA	This work
RBS_{B0034}	AAAGAGGAGAAA	Parts Registry: BBa_B0034
RBS_{500N}	TAGAAAGACTTACTTAATAACATTCTTTTTCTAAT	pTSara-N (Shis *et al.*)[6]
RBS_{500C}	GGAGCCCTATATATAAAGATCAGGGCCAGGT	pTSara-C (Shis *et al.*)[6]
RBS_{B0033}	TCACACAGGAC	Parts Registry: BBa_B0033
RBS_{S175}	GGATATTAACTAATTTAACGGCATCACGAGTA	pR33S175 (Grant *et al.*)[3]
RBS_{250}	GGCTAGTTCCAACGAACAAAAACCCAATACAAAC	This work
RBS_{100}	CAAGGGTTCAGAGAAGCAACTTTACCACTTCCT	This work
RBS_{50}	CCATACATTATTCTCGCAAGTCCAGAATAGCGT	This work

Supplementary Table 3 | Regulatory elements used in this work. Compared to P_{J23101}, the $P_{J23101*}$ promoter included an A-to-T point mutation in position 25, which spontaneously arose during plasmid construction. To test the impact of this mutation, we constructed a ratiometric reporter plasmid p1cVTm where expression of *mVenus* was controlled by $P_{J23101*}$, expression of *mTurquoise2* by P_{J23101}. Ratiometric analysis of p1cVTm in *TOP10 E. coli* showed $P_{J23101*}$ to outperform P_{23101} in relative promoter activity by 1.4 ± 0.1-fold.

Plasmid Name (Genbank ID)	Plasmid Contents	Source	Description
p1cVTm (KY643824)	*pSB1A3*:$P_{J23101*}$-RBS_{B0034}-*mVenus*, P_{J23101}-RBS_{B0034}-*mTurquoise2; AmpR; pMB1 ori*	This work	Ratiometric control plasmid to assess $P_{J23101*}$
p1wtVT-lacI (KX986152)	*pSB1A3*:P_{T7}-RBS_{B0034}-*mVenus*, P_{J23101}-RBS_{B0034}-*lacI(LVA)*, P_{J23101}-RBS_{B0034}-*mTurquoise2; AmpR; pMB1 ori*	This work	Ratiometric reporter [P_{T7}-RBS_{B0034}, high copy number]
p4wtVT-lacI (KX986153)	*pSB4A5*:P_{T7}-RBS_{B0034}-*mVenus*, P_{J23101}-RBS_{B0034}-*lacI(LVA)*, P_{J23101}-RBS_{B0034}-*mTurquoise2; AmpR; pSC101 ori*	This work	Ratiometric reporter [P_{T7}-RBS_{B0034}, low copy number]
p1g3VT-lacI (KX986154)	*pSB1A3*:P_{T7}*(-3G)*-RBS_{B0034}-*mVenus*, P_{J23101}-RBS_{B0034}-*lacI(LVA)*, P_{J23101}-RBS_{B0034}-*mTurquoise2; AmpR; pMB1 ori*	This work	Ratiometric reporter [P_{T7}(-3G)-RBS_{B0034}, high copy number]
p4g3VT-lacI (KX986155)	*pSB4A5*:P_{T7}*(-3G)*-RBS_{B0034}-*mVenus*, P_{J23101}-RBS_{B0034}-*lacI(LVA)*, P_{J23101}-RBS_{B0034}-*mTurquoise2; AmpR; pSC101 ori*	This work	Ratiometric reporter [P_{T7}(-3G)-RBS_{B0034}, low copy number]
p1enVT-lacI (KX986156)	*pSB1A3*:P_{T7}-RBS_{T7g10}-*mVenus*, P_{J23101}-RBS_{B0034}-*lacI(LVA)*, P_{J23101}-RBS_{B0034}-*mTurquoise2; AmpR; pMB1 ori*	This work	Ratiometric reporter [P_{T7}-RBS_{T7g10}, high copy number]
p4enVT-lacI (KX986157)	*pSB4A5*:P_{T7}-RBS_{T7g10}-*mVenus*, P_{J23101}-RBS_{B0034}-*lacI(LVA)*, P_{J23101}-RBS_{B0034}-*mTurquoise2; AmpR; pSC101 ori*	This work	Ratiometric reporter [P_{T7}-RBS_{T7g10}, low copy number]
p1eng3VT-lacI (KX986158)	*pSB1A3*:P_{T7}*(-3G)*-RBS_{T7g10}-*mVenus*, P_{J23101}-RBS_{B0034}-*lacI(LVA)*, P_{J23101}-RBS_{B0034}-*mTurquoise2; AmpR; pMB1 ori*	This work	Ratiometric reporter [P_{T7}(-3G)-RBS_{T7g10}, high copy number]
p4eng3VT-lacI (KX986159)	*pSB4A5*:P_{T7}*(-3G)*-RBS_{T7g10}-*mVenus*, P_{J23101}-RBS_{B0034}-*lacI(LVA)*, P_{J23101}-RBS_{B0034}-*mTurquoise2; AmpR; pSC101 ori*	This work	Ratiometric reporter [P_{T7}(-3G)-RBS_{T7g10}, low copy number]
p4g3VT (KX986160)	*pSB4A5*:P_{T7}*(-3G)*-RBS_{B0034}-*mVenus*, P_{J23101}-RBS_{B0034}-*mTurquoise2; AmpR; pSC101 ori*	This work	Ratiometric reporter [P_{T7}(-3G)-RBS_{B0034}, low copy number, LacI generator removed]
pTara:500	*pBAD33*:P_{araC}-*araC*, P_{BAD}-RBS_{500N}-*T7RNAP; CmR; p15A ori*	Shis *et al.* [6]	Arabinose-inducible T7RNAP
pTara:500* (KX986161)	*pBAD33*:P_{araC}-*araC*, P_{BAD}-RBS_{500N}-*T7*RNAP; CmR; p15A ori*	This work	Arabinose-inducible T7*RNAP
pTSara-N	*pBAD33*:P_{araC}-*araC*, P_{BAD}-RBS_{500N}-*T7RNAP[1-179]; CmR; p15A ori*	Shis *et al.* [6]	Arabinose-inducible T7RNAP[1-179]
pTSara-C	*pBAD33*:P_{araC}-*araC*, P_{BAD}-RBS_{500C}-*T7RNAP[180-880]; CmR; p15A ori*	Shis *et al.* [6]	Arabinose-inducible T7RNAP[180-880]
pTSara	*pBAD33*:P_{araC}-*araC*, P_{BAD}-RBS_{500N}-*T7RNAP[1-179]*, P_{BAD}-RBS_{500C}-*T7RNAP[180-880]; CmR; p15A ori*	Shis *et al.* [6]	Arabinose-inducible split T7RNAP

pTSara* (KX986162)	*pBAD33:*P_{araC}*-araC,* P_{BAD}*-*RBS_{500N}*-T7RNAP[1-179],* P_{BAD}*-*RBS_{500C}*-T7*RNAP[180-880]; CmR; p15A ori*	This work	Arabinose-inducible split T7*RNAP
pTSaraSZ (KX986163)	*pBAD33:*P_{araC}*-araC,* P_{BAD}*-*RBS_{500N}*-T7RNAP[1-179]-SynZIP17,* P_{BAD}*-*RBS_{500C}*-SynZIP18-T7RNAP[180-880]; CmR; p15A ori*	This work	Arabinose-inducible split T7RNAP with SynZIP domains 17/18
pTSara*SZ (KX986164)	*pBAD33:*P_{araC}*-araC,* P_{BAD}*-*RBS_{500N}*-T7RNAP[1-179]-SynZIP17,* P_{BAD}*-*RBS_{500C}*-SynZIP18-T7*RNAP[180-880]; CmR; p15A ori*	This work	Arabinose-inducible split T7*RNAP with SynZIP domains 17/18
p4g3R (KX986165)	*pSB4A5:*P_{T7}*(-3G)-*RBS_{B0034}*-mRFP1; AmpR; pSC101 ori*	This work	RFP reporter [P_{T7}(-3G)-RBS_{B0034}, low copy number]
pCRB DRT7VTPLux*500 (KX986166)	*pR33S175:*P_{R0040}*-*RBS_{B0033}*-luxR,* P_{R0011}*-*RBS_{S175}*-lasR,* P_{Las81*}*-*RBS_{B0033}*-T7RNAP[1-179]-*RBS_{B0034}*-mVenus,* P_{Lux76*}*-*RBS_{B0033}*-T7RNAP[180-880]-*RBS_{B0034}*-mTurquoise2; KanR; p15A ori*	This work	Controller plasmid [ca. 558.5 relative RBS strength at P_{Las81*}]
pCRB DRT7VTPLux*500CFP (KX986167)	*pR33S175:*P_{R0040}*-*RBS_{B0033}*-luxR,* P_{R0011}*-*RBS_{S175}*-lasR,* P_{Lux76*}*-*RBS_{B0033}*-T7RNAP[180-880]-*RBS_{B0034}*-mTurquoise2; KanR; p15A ori*	This work	CFP half-circuit controller plasmid [ca. 558.5 relative RBS strength at P_{Las81*}]
pCRB DRT7VTPLux*500YFP (KX986168)	*pR33S175:*P_{R0040}*-*RBS_{B0033}*-luxR,* P_{R0011}*-*RBS_{S175}*-lasR,* P_{Las81*}*-*RBS_{B0033}*-T7RNAP[1-179]-*RBS_{B0034}*-mVenus; KanR; p15A ori*	This work	YFP half-circuit controller plasmid [ca. 558.5 relative RBS strength at P_{Las81*}]
pCRB DRT7VTPLux*250 (KX986169)	*pR33S175:*P_{R0040}*-*RBS_{B0033}*-luxR,* P_{R0011}*-*RBS_{S175}*-lasR,* P_{Las81*}*-*RBS_{250}*-T7RNAP[1-179]-*RBS_{B0034}*-mVenus,* P_{Lux76*}*-*RBS_{B0033}*-T7RNAP[180-880]-*RBS_{B0034}*-mTurquoise2; KanR; p15A ori*	This work	Controller plasmid [ca. 249.0 relative RBS strength at P_{Las81*}]
pCRB DRT7VTPLux*100 (KX986170)	*pR33S175:*P_{R0040}*-*RBS_{B0033}*-luxR,* P_{R0011}*-*RBS_{S175}*-lasR,* P_{Las81*}*-*RBS_{100}*-T7RNAP[1-179]-*RBS_{B0034}*-mVenus,* P_{Lux76*}*-*RBS_{B0033}*-T7RNAP[180-880]-*RBS_{B0034}*-mTurquoise2; KanR; p15A ori*	This work	Controller plasmid [ca. 110.5 relative RBS strength at P_{Las81*}]
pCRB DRT7VTPLux*50 (KX986171)	*pR33S175:*P_{R0040}*-*RBS_{B0033}*-luxR,* P_{R0011}*-*RBS_{S175}*-lasR,* P_{Las81*}*-*RBS_{50}*-T7RNAP[1-179]-*RBS_{B0034}*-mVenus,* P_{Lux76*}*-*RBS_{B0033}*-T7RNAP[180-880]-*RBS_{B0034}*-mTurquoise2; KanR; p15A ori*	This work	Controller plasmid [ca. 47.0 relative RBS strength at P_{Las81*}]
pSB1C3 I0500 LuxI (KX986172)	*pSB1C3:*P_{araC}*-araC,* P_{BAD}*-luxI; CmR; pMB1 ori*	This work	Arabinose-inducible 3OC6HSL sender plasmid
pSB1C3 I0500 LasI (KX986173)	*pSB1C3:*P_{araC}*-araC,* P_{BAD}*-lasI; CmR; pMB1 ori*	This work	Arabinose-inducible 3OC12HSL sender plasmid

Supplementary Table 4 | Plasmids used in this work.

Supplementary References

1. Salis, H. M. The ribosome binding site calculator. *Methods Enzym.* **498,** 19–42 (2011).

2. Rosenberg, A. H. *et al.* Vectors for selective expression of cloned DNAs by T7 RNA polymerase. *Gene* **56,** 125–135 (1987).

3. Grant, P. K. *et al.* Orthogonal intercellular signaling for programmed spatial behavior. *Mol. Syst. Biol.* **12,** 849–849 (2016).

4. Federici, F., Dupuy, L., Laplaze, L., Heisler, M. & Haseloff, J. Integrated genetic and computation methods for in planta cytometry. *Nat. Methods* **9,** 483–485 (2012).

5. Goedhart, J. *et al.* Structure-guided evolution of cyan fluorescent proteins towards a quantum yield of 93%. *Nat. Commun.* **3,** 751 (2012).

6. Shis, D. L. & Bennett, M. R. Library of synthetic transcriptional AND gates built with split T7 RNA polymerase mutants. *Proc. Nat. Acad. Sci. USA* **110,** 5028–5033 (2013).
